# Tryptophan degradation by intestinal *Bacteroides* induces anti-tumor immunity and limits melanoma growth

**DOI:** 10.1016/j.xcrm.2026.102921

**Published:** 2026-07-14

**Authors:** Ximena Diaz Olea, Kristin Beede, Gabriel Pereira, David Scott, Christopher Petucci, Eric Martens, Dmitri Rodionov, Aagam Shah, Miguel P. Martinez, Hyungsoo Kim, Ashok Kumar Sharma, Anthony Martin, Tongwu Zhang, Mark B. Faries, Omid Hamid, Suzanne Devkota, Andrei Osterman, Simon Knott, Emile E. Voest, Nadim J. Ajami, Jennifer Wargo, Amanda E. Ramer-Tait, Ze’ev A. Ronai

**Affiliations:** 1 Translational Research Institute, Elinor and Rendall Department of Surgery, Cedars Sinai Medical Center, Los Angeles, CA 90048, USA; 2 Department of Biomedical Sciences, Cedars Sinai Medical Center, Los Angeles, CA 90048, USA; 3 Sanford Burnham Prebys Medical Discovery Institute, La Jolla, CA 92037, USA; 4 Department of Food Science and Technology, Nebraska Food for Health Center, University of Nebraska-Lincoln, Lincoln, NE 68588, USA; 5 Department of Microbiology & Immunology, University of Michigan Medical School, Ann Arbor, MI 48109, USA; 6 Perelman School of Medicine, University of Pennsylvania, Philadelphia, PA 19104, USA; 7 Division of Molecular Oncology & Immunology, the Netherlands Cancer Institute, Amsterdam 1066 CX, the Netherlands; 8 Human Microbiome Research Institute, Cedars Sinai Medical Center, Los Angeles, CA 90048, USA; 9 Division of Cancer Epidemiology & Genetics, National Cancer Institute, Rockville, MD 20892, USA; 10 The Angeles Clinic and Research Institute, Cedars Sinai Medical Center, Los Angeles, CA 90025, USA; 11 Department of Genomic Medicine, The University of Texas MD Anderson Cancer Center, Houston, TX 77030, USA

**Keywords:** tryptophan, *Bacteroides uniformis*, *Bacteroides rodentium*, melanoma, indoles, anti-tumor immunity

## Abstract

Study of gut microbiota control of anti-tumor immunity (ATI) identifies *Bacteroides rodentium* and the human-related *Bacteroides uniformis* species to be capable of inducing ATI and limiting melanoma development in germ-free (GF), complex microbiome, or wild-type (WT) mice. Enhanced CD8^+^ T cell infiltration within tumors of mice harboring *B. rodentium* coincides with increased expression of immune-stimulating pathways. Metabolomic analyses identify lower tryptophan levels in the cecal samples of GF mice harboring *B. rodentium*. *In silico* genomic reconstruction reveals that *B. rodentium* and *B. uniformis* harbor tryptophanase A (*TnaA*) and aromatic aminotransferase genes, which degrade tryptophan to indoles. Administration of *B. uniformis* harboring *TnaA* mutant fails to inhibit melanoma growth. Notably, administration of indoles effectively induces ATI and inhibits melanoma development. Correspondingly, the levels of bacterially encoded tryptophan-degrading enzymes are higher in cohorts of patients with melanoma responding to immunotherapy. These findings identify indoles as tryptophan breakdown products capable of inducing ATI resulting in melanoma inhibition.

## Highlights

• Tryptophan degradation by *B. rodentium/B. uniformis* enzymes induces anti-tumor immunity
• *TnaA* degrades tryptophan to indoles, which inhibit melanoma
• Indole induces immune cell infiltration into melanoma, inhibiting their growth
• Increase in abundance of *TnaA* and *ArAT* seen in responders to ICB

## Graphical abstract

**Figure:**
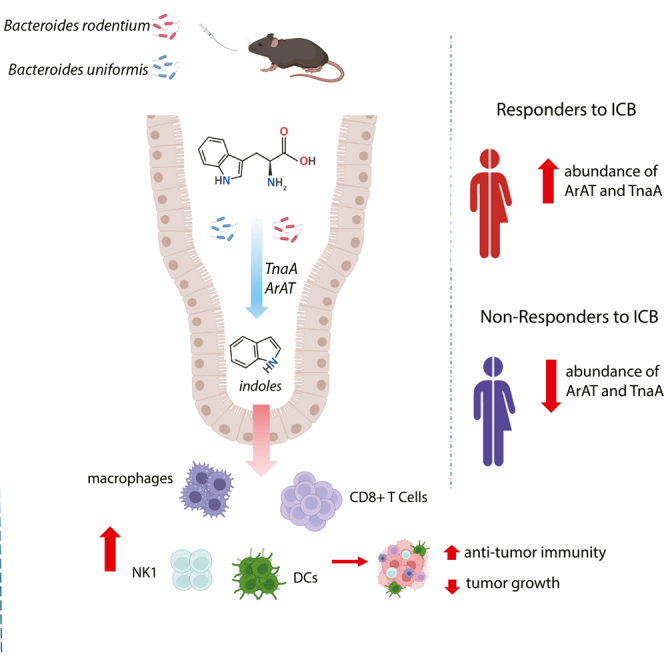

## Teaser

Diaz-Olea et al. identify indoles as tryptophan breakdown products that induce anti-tumor immunity and inhibit melanoma growth. Indoles, produced upon tryptophan breakdown by *Bacteroides uniformis-* (human) or *Bacteroides rodentium-* (mouse) encoded enzymes, induce anti-tumor immunity independent of AhR signaling. Patients who respond to ICB exhibit higher expression of these enzymes.

## Introduction

Melanoma, which exhibits high metastatic propensity and resistance to therapy, is one of the most aggressive skin cancers. Although combining immunotherapy with targeted therapies holds great promise against melanoma,^1^^,^^2^ alternatives that overcome immune evasion and enable immune system recognition of immune-cold tumors remain an unmet need. Among options to address these needs are strategies to selectively alter the gut microbiota, which play an important role in controlling melanoma development and enhancing responses to chemotherapy, radiation, or immunotherapies.^3^^,^^4^^,^^5^^,^^6^^,^^7^ In mouse models, strategies to enrich for specific gut bacteria, such as *Bacteroides* in melanoma^4^ or *Faecalibaculum* and *Bacteroides* in colorectal cancer (CRC),^8^ reportedly attenuate tumor growth, although the underlying mechanisms remain unknown.

Efforts to define the mechanisms regulating anti-tumor immunity led us to investigate RNF5, a membrane-anchored E3 ubiquitin ligase that functions in endoplasmic reticulum-associated protein degradation (ERAD).^9^ RNF5 serves to clear misfolded proteins such as CFTR, SLC1A5, and S100A8, which are implicated in the pathogenesis of multiple chronic diseases, including inflammatory bowel disease and cancer.^10^^,^^11^^,^^12^ Notably, we found that *Rnf5*-knockout (KO) mice exhibit limited tumor growth due to enhanced anti-tumor immunity driven by changes in the gut microbiota.^4^ Administration of 11 bacterial strains enriched in *Rnf5* KO mice was sufficient to induce anti-tumor immunity and inhibit melanoma growth in germ-free (GF) mice.^4^

The mechanisms underlying the regulation of anti-tumor immunity are under intense investigation. Growing evidence indicates that metabolic activities play a key role in anti-tumor immunity, with particular attention to the amino acid tryptophan, which may antagonize anti-tumor immunity.^13^^,^^14^^,^^15^ Notably, analysis of this activity has largely focused on cellular changes in both cancer and immune cells, although the possible role of a more systemic cause remains plausible. In humans and mice, tryptophan must be obtained exclusively through diet. Tryptophan is also a precursor to diverse metabolites produced by numerous intestinal microorganisms^14^ that have been implicated in intestinal immune tolerance and gut microbiome homeostasis.^13^^,^^14^^,^^15^ Numerous studies report that certain bacterial species can also activate subsets of immune cells, either directly or indirectly, resulting in varying degrees of anti-tumor immune activity.^5^^,^^16^^,^^17^^,^^18^ The amino acid tryptophan is implicated in these activities and has been shown to negatively regulate immune system factors and block anti-tumor immunity, largely by regulating the activity of the aryl hydrocarbon receptor (AhR) and indoleamine 2, 3-dioxygenase 1 (IDO1).^19^^,^^20^^,^^21^

Here, we aimed to define specific gut bacterial species and related metabolites required for anti-tumor immunity and melanoma growth inhibition using mouse models with varying microbiome complexity.^22^^,^^23^ Our analysis identified *Bacteroides rodentium,* one of the 11 bacterial strains we previously reported to be enriched in the gut microbiome of *Rnf5* KO mice*,* and a closely related species found in humans, *Bacteroides uniformis,* as capable of enhancing anti-tumor immunity and limiting melanoma as well as growth of other tumor types. Deletion of *tryptophanase A* (*TnaA*), which encodes an enzyme catalyzing tryptophan degradation in *B. uniformis*, blocked induction of anti-tumor immunity and its ability to inhibit melanoma growth. Correspondingly, the level of tryptophan-degrading enzymes increases in patients who respond to immune checkpoint blockade (ICB). Collectively, these studies establish that tryptophan degradation by the gut microbiota is an important process in controlling anti-tumor immunity and tumor growth.

## Results

### *B. rodentium* induces anti-tumor immunity and inhibits melanoma growth

**Figure 1:**
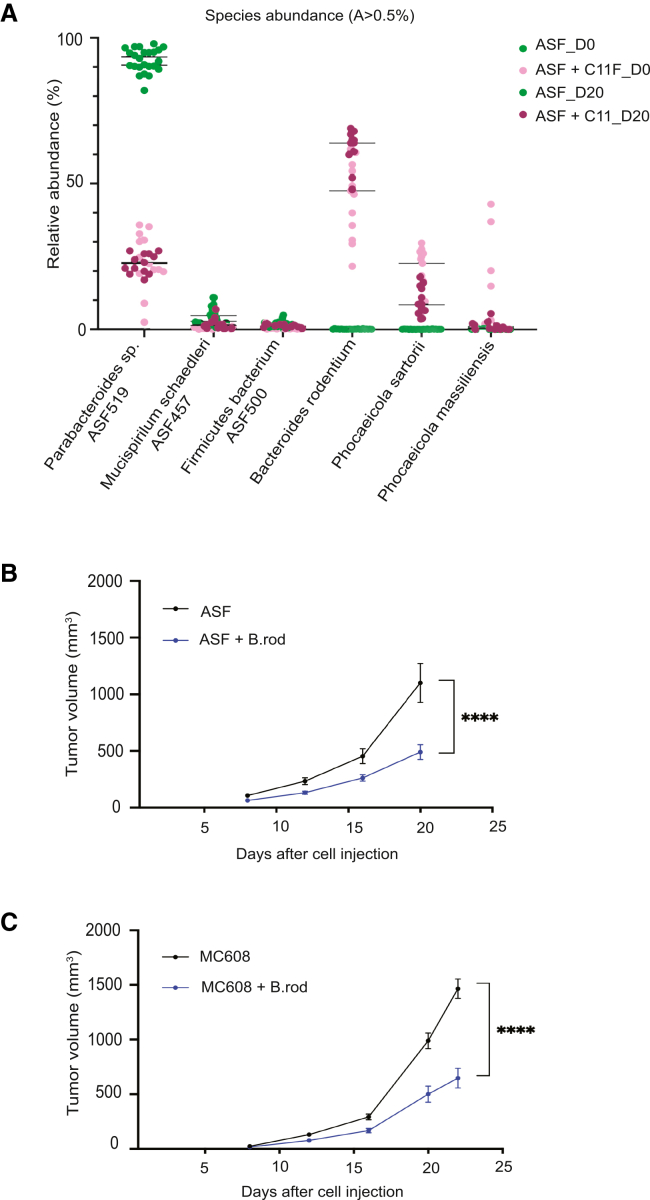
*B. rodentium* limited tumor growth (A) Average relative abundance (A%) >0.5% of bacterial species in the gut microbiomes of germ-free (GF) mice administered the altered Schaedler flora (ASF) or ASF plus a cocktail of 11 bacterial species on days 0 and 20 days after subcutaneous injection of YUMM 1.5 cells. (B) Tumor growth in GF mice colonized with either ASF or ASF plus *B. rodentium* via oral gavage 14 days prior to YUMM1.5 tumor cell injection (*n* = 15 mice/treatment; data represent two experiments). (C) Tumor growth in GF mice colonized with either microbiome MC608-F-a1 or MC608-F-a1 plus *B. rodentium* 14 days prior to YUMM1.5 tumor cell injection (*n* = 18 mice/treatment; data represent two experiments). All data were analyzed by unpaired *t* test. Data are represented as mean ± SEM; ∗∗∗∗*p* < 0.0001 using two-way ANOVA. See also Figure S1.

Our earlier studies identified 11 bacterial species that enhanced anti-tumor immunity and inhibited melanoma development when provided to GF mice harboring the altered Schaedler flora (ASF).^4^ Analyses identified three bacterial strains—*B. rodentium, Phocaicola sartorii*, and *Phocaicola massiliensis*—that were significantly enriched at the end of this study (tumor collection point) (Figure 1A). Of those, colonization of *B. rodentium* with ASF promoted immune development in GF recipients, inhibiting melanoma growth as evidenced by decreased tumor size compared with ASF controls (Figures 1B; Figure S1C). However, administering *Phocaeicola sartorii* and *Phocaeicola massiliensis* abrogated anti-tumor effects mediated by *B. rodentium* (Figures S1A and S1B). Importantly, colonizing MC608-F-a1 mice harboring a more complex microbiota deficient in *Bacteroides*^22^^,^^23^^,^^24^ with *B. rodentium* was also sufficient to inhibit melanoma growth (Figures 1C; Figure S1D). Notably, the presence of the *Bacteroides* genus was associated with better overall survival in The Cancer Genome Atlas (TCGA) solid-tumor cohort (Figure S1E).

### *B. rodentium*-colonized mice show immune cell infiltration of tumors

**Figure 2:**
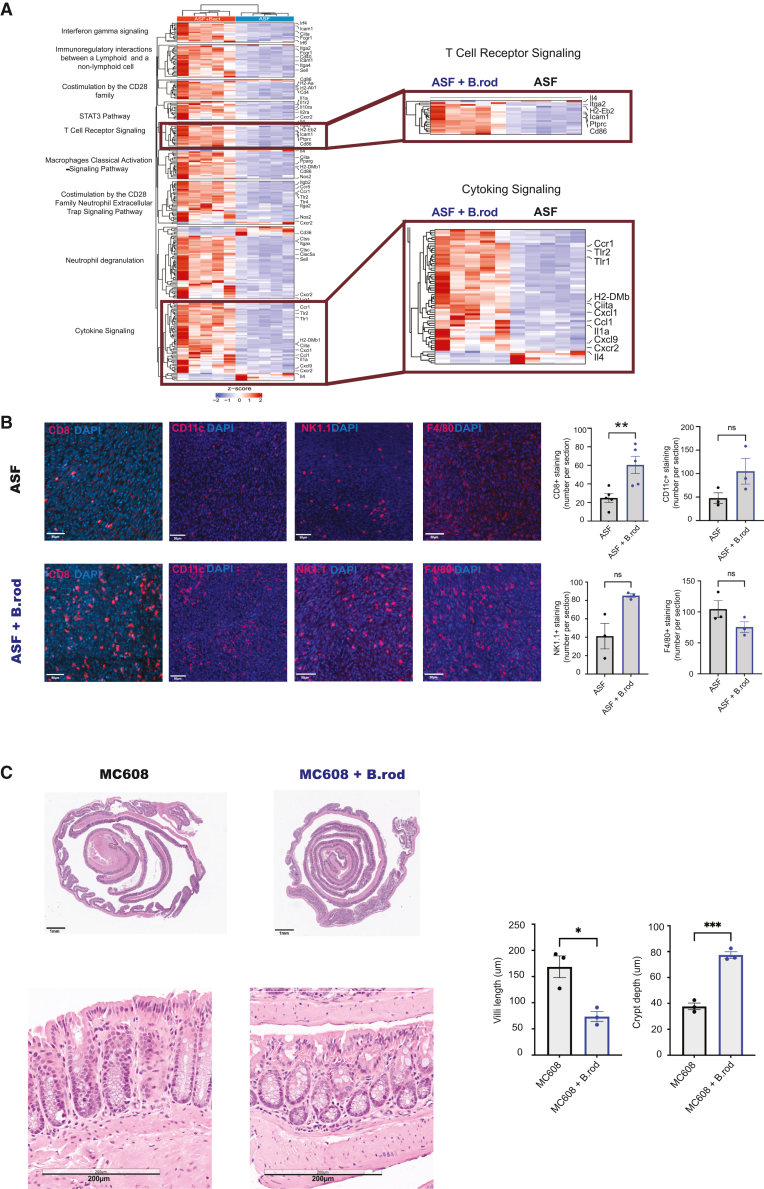
Upregulated immune signaling in mice colonized with *B. rodentium* Gene expression changes were assessed by RNA-seq (on day 22) in tumor samples from GF mice colonized with ASF or ASF plus *B. rodentium* (*n* = 6 mice/treatment; two independent experiments). (A) Bioinformatic analysis following RNA-seq was performed on tumors from GF mice colonized with ASF or ASF plus *B. rodentium*. Heatmap shows differentially expressed pathways. (B) Staining with anti-mouse CD8, CD11c, NK1.1, and F4/80 antibodies of tumor sections described above. CD8^+^, CD11c^+^, NK1.1^+^, and F4/80^+^ cells were quantified (*n* = 3 mice/treatment in two experiments). For image analysis, 3 images were randomly selected from different areas of each tumor, in which the number of positive cells per section were counted. Presented is the average number of these three sections. Scale bars, 50 μm. (C) Staining with H&E of Swiss roll sections from small intestines obtained from mice colonized with MC608-Fa1 or MC608-Fa1 plus *B. rodentium.* Villi lengths and crypt depth were measured (*n* = 3 mice/treatment in two experiments). Scale bars: 900 μm for top images and 200 μm for bottom images. All data were analyzed by unpaired *t* test. Data are represented as mean ± SEM, ns, not significant; ∗*p* < 0.05, ∗∗*p* < 0.005, ∗∗∗*p* < 0.001, using two-way ANOVA or Mann-Whitney U test. See also Figure S2 and Table S1.

To assess gene expression changes associated with anti-tumor immunity, we performed RNA sequencing (RNA-seq) of tumor tissues collected from GF mice colonized with ASF in the presence or absence of *B. rodentium*. Notably, relative to controls receiving only ASF, mice harboring *B. rodentium* exhibited enhanced expression of genes associated with immune activation (Figure 2A), including CD86 and ICAM (implicated in T cell receptor signaling); CCR1, TLR1, and TLR2 (implicated in cytokine signaling); and several cytokines, including TNFα and IFNγ (Figures 2A; Figures S2A and S2B). Quantitative reverse-transcription PCR (RT-qPCR) analysis confirmed the upregulation of these genes in the tumors obtained from mice colonized with *B. rodentium* versus controls (Figures S2A and S2B). Further analysis showed enhanced CD8^+^ T cell infiltration of the tumors obtained from mice colonized with *B. rodentium* compared with ASF controls (Figure 2B). To test whether *B. rodentium* alters the gut epithelial barrier, we examined the architecture of the small intestine in MC608-F-a1 mice colonized with *B. rodentium.* We noted shorter villi and increased number of crypts in mice colonized with *B. rodentium* compared with MC608-F-a1 controls (Figure 2C), similar to outcomes seen in mice that were previously reported to exhibit enhanced anti-tumor immunity.^4^

To assess how *B. rodentium* impacts immune cell function, we collected the secretome from *in vitro* cultures of *B. rodentium* and incubated it with bone marrow-derived dendritic cells (BMDCs) from naive mice. BMDCs exposed to the *B. rodentium* secretome showed increased expression of the markers of dendritic cell activation such as CD80, CD40, and MHC I relative to untreated BMDCs (Figure S2C). These observations suggest that *B. rodentium* can activate immune cells that function in anti-tumor immunity.

### Tryptophan depletion inhibits melanoma growth

**Figure 3:**
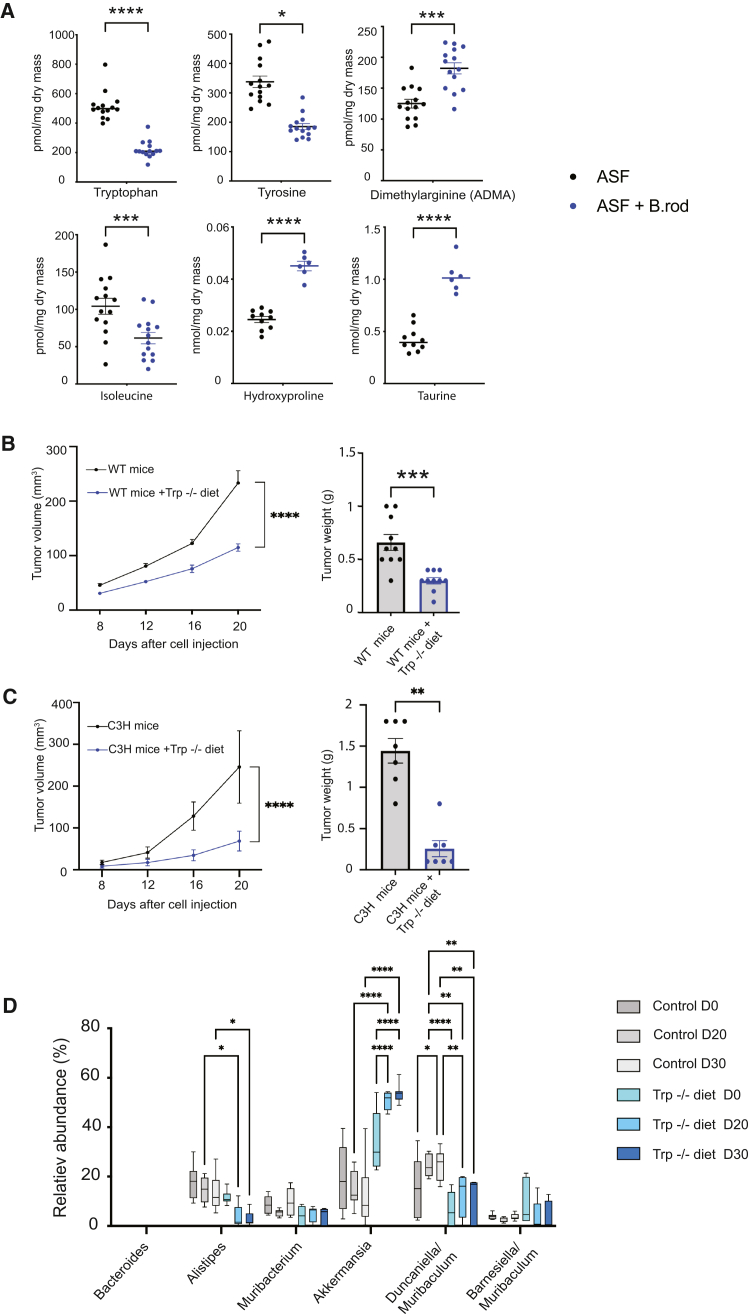
Lower levels of tryptophan were identified in metabolomic analysis of cecal samples from gnotobiotic mice (A) Quantification of indicated metabolites in cecal samples from GF mice colonized with ASF or ASF plus *B. rodentium* (*n* = 6 mice/treatment). (B and C) Growth and weight of tumors in conventional C57BL/6J mice (B) and C3H mice (C) provided with the indicated diets (control or tryptophan deficient) 3 days prior to YUMM1.5 tumor cell injection (day 0; *n* = 10 mice/treatment). (D) Taxonomic phenotype profile of bacterial strains was obtained at the indicated time points from fecal samples. Analyses were based on 16S rRNA bacterial gene sequencing followed by bioinformatic analyses. All data were analyzed by unpaired *t* test. Represented as mean ± SEM; ∗*p* < 0.05, ∗∗*p* < 0.005, ∗∗∗*p* < 0.001, ∗∗∗∗*p* < 0.0001 using two-tailed *t* test or two-way ANOVA. See also Figure S3 and Tables S2 and S3.

To assess the mechanisms underlying anti-tumor activity by *B. rodentium*, we performed metabolomic analysis of cecal samples from mice colonized with ASF plus *B. rodentium* compared with ASF-only controls (Table S3). Strikingly, we observed significantly reduced levels of tryptophan in cecal samples of mice colonized with *B. rodentium* plus ASF compared with ASF only (Figure 3A). Mice harboring *B. rodentium* also showed decreased cecal levels of tyrosine and isoleucine and increased levels of the host-derived metabolites dimethylarginine, hydroxyproline, and taurine (Figure 3A). Independent metabolomic analyses confirmed increased metabolites associated with tryptophan metabolism (Figure S3A). Consistent with these observations, changes in the level of tryptophan metabolites produced by the bacterial enzymes were among the more pronounced (Figure S3B). Correspondingly, elevated levels of tryptophan degradation products, such as quinolinic acid and kynurenic acid, which are derived upon tryptophan degradation by the host, were also identified (Figure S3C). Supplementing the diet of conventional C57BL/6J mice with either kynurenic acid or taurine, both previously shown to affect anti-tumor immunity in independent studies,^25^^,^^26^ did not alter tumor development (Figures S3D and S3E). Further, conventional C57BL/6J or C3H mice fed a tryptophan-deficient diet starting 3 days prior to tumor cell injection showed significant inhibition of melanoma development compared with mice fed a diet containing tryptophan (Figures 3B and 3C). Notably, these mice also showed marked changes in gut bacterial composition (Figure S3F), the most notable being a significant enrichment in *Akkermansia muciniphila* (Figure 3D), a tryptophan prototroph strain previously associated with positive responses in patients to immune checkpoint therapies.^27^ At the same time, the relative abundance of *B. rodentium* was unaltered (Figure S3G), suggesting that changes elicited upon tryptophan omission from the diet are not mediated by *B. rodentium*. Altogether, these results point to the importance of multiple bacterial strains in tumor inhibition and highlight the importance of tryptophan degradation in tumor growth control.

### *B. uniformis* phenocopies the effects of *B. rodentium*

**Figure 4:**
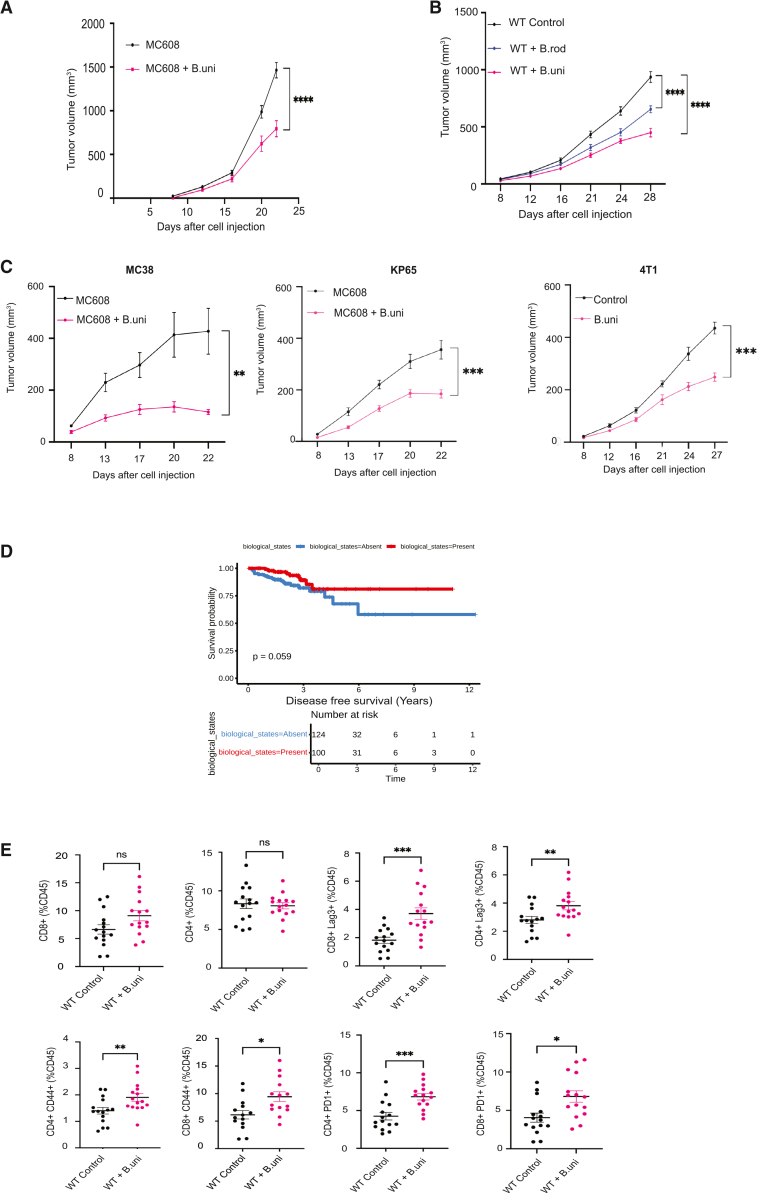
*B. uniformis* colonization phenocopied the tumor inhibition seen upon *B. rodentium* colonization (A) Tumor growth in GF mice colonized with either microbiome MC608-F-a1 or MC608-F-a1 plus *B. uniformis* via oral gavage three times a week, starting 14 days prior to subcutaneous injection with YUMM1.5 cells (*n* = 8 mice/treatment). (B) Tumor growth in conventional C57BL/6J mice that were colonized with either *B. rodentium or B. uniformis* via oral gavage three times a week, starting 14 days prior to subcutaneous injection with YUMM1.5 cells (*n* = 7 mice/treatment). (C) Tumor growth in mice colonized with either MC608-F-a1 or MC608-F-a1 plus *B. uniformis* via oral gavage three times a week, starting 14 days prior to subcutaneous injection with MC38 cells or KP65 cells (*n* = 8 mice/treatment), and tumor growth in conventional BALB/c mice that were colonized with *B. uniformis* via oral gavage three times a week, starting 14 days prior to subcutaneous injection with 4T1 cells (*n* = 8 mice/treatment). (D) Kaplan-Meier analysis of % survival (disease-free survival, years) relative to the abundance of *B. uniformis.* Indicated are the biological states (presence or absence of the indicated bacterial species). The log rank test *p* value is indicated in the survival plot. Cox proportional hazards model results: hazard ratio = 0.50 (95% confidence interval: 0.22–1.12), *p* = 0.09. (E) Quantification of tumor infiltration of CD8^+^ and CD4^+^ T cells and CD44^+^, Lag 3^+^, and PD1^+^ on CD4^+^ CD8^+^ T cell 12 days after injection of YUMM1.5 cells into conventional C57BL/6J mice that were colonized with *B. uniformis* (via oral gavage three times a week), starting 14 days prior to tumor inoculation until end of the experiment (*n* = 15 mice/treatment). All data were analyzed by unpaired *t* test. Represented as mean ± SEM; ns, not significant; ∗*p* < 0.05, ∗∗*p* < 0.005, ∗∗∗*p* < 0.001, ∗∗∗∗*p* < 0.0001 using two-tailed *t* test or two-way ANOVA. See also Figure S4 and Tables S4 and S5.

*In silico* genomic reconstruction of metabolic pathways relevant to the 11 species of microbes enriched in *Rnf5* KO mice revealed *B. rodentium* to be the only strain whose genome harbored the tryptophanase (*TnaA*) gene. As noted above, *TnaA* encodes an enzyme that degrades tryptophan. Given that others have linked tryptophan to the inhibition of anti-tumor immunity,^19^^,^^28^^,^^29^ we searched for *TnaA*-expressing bacterial species, particularly those found in the human gut. For these analyses, we sought species or strains that also harbored *ArAT*, which encodes an enzyme also functioning in tryptophan degradation. In this effort, we identified *B. uniformis*, a species that expresses a *TnaA* ortholog and *ArAT* and is commonly found in the human gastrointestinal tract. When compared with other *Bacteroides* strains, *B. uniformis* is phenotypically and genetically close to *B. rodentium* (Tables S4, and S5). Administration of *B. uniformis* recapitulated the changes observed with *B. rodentium* in inhibiting melanoma growth in GF mice as well as in mice harboring more complex gut microbiome, including GF mice colonized with a microbiota deficient in *Bacteroides* (MC608-F-a1), and in conventional C57BL/6J mice (Figures 4A and 4B; Figures S4A and S4B). Notably, *B. uniformis* administration also inhibited the growth of CRC, pancreatic cancer in MC608-F-a1 mice, and breast cancer in conventional Balb/c mice (Figure 4C). Notably, TCGA analysis revealed that the presence of *B. uniformis* was also associated with better remission in solid tumors (Figure 4D).

We next determined whether *B. uniformis* induces changes in factors or pathways associated with immune activity, phenotypes we have previously observed with *B. rodentium*. RT-qPCR analyses of RNA derived from melanoma tumors subjected to treatment with the indicated bacterial strains indicated that *B. uniformis* colonization promoted notable increases in the expression of markers associated with pathogen-induced cytokine signaling pathways in CCR1-, TLR1- and TLR2-positive immune cells (Figures S4C and S4D). We then used immunophenotyping to assess the anti-tumor responses 12 days after melanoma tumor cell inoculation into conventional C57BL/6J mice. We performed fluorescence-activated cell sorting (FACS) of tumor-infiltrated immune cells on CD45^+^-enriched cell populations and identified increased infiltration of CD44^+^ CD8^+^ and CD4^+^ T cells into tumors isolated from mice colonized with *B. uniformis* compared with controls in conventional C57BL/6J mice. These findings coincided with increased expression of the checkpoint receptors PD1 and Lag3 on CD8^+^ and CD4^+^ T cells (Figure 4E). We observed no changes in the expression of markers associated with dendritic cells, macrophages, or natural killer cells after *B. uniformis* administration (data not shown). Altogether, these results suggest that *B. uniformis,* like *B. rodentium*, promotes anti-tumor immunity.

### The enzyme encoded by *TnaA* gene is required for *B. uniformis* to inhibit melanoma growth

**Figure 5:**
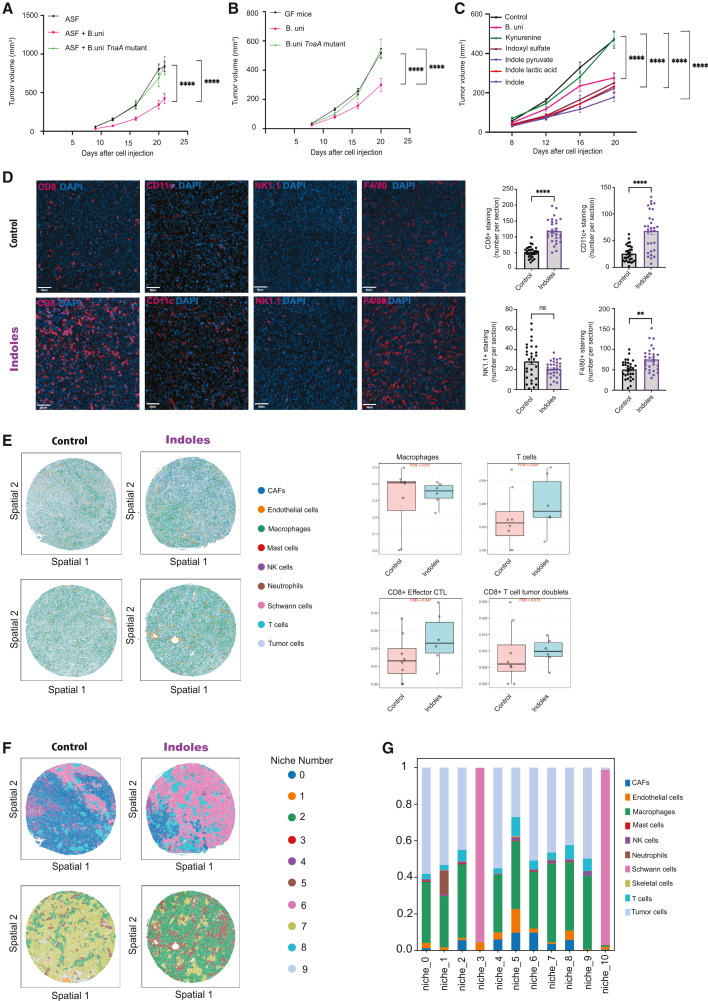
*B. uniformis* colonization phenocopying tumor inhibition seen upon *B. rodentium* colonization requires the tryptophanase (*TnaA*) gene (A) Tumor growth in GF mice colonized with ASF, ASF plus *B. uniformis,* or ASF plus *B. uniformis* tryptophanase mutant 14 days prior to YUMM1.5 tumor cell injection (*n* = 15 mice/treatment). (B) Tumor growth in GF mice colonized with either *B. uniformis* or *B. uniformis TnaA* mutant 14 days prior to YUMM1.5 tumor cell injection (*n* = 15 mice/treatment). (C) Tumor growth in conventional C57BL/6J mice that were treated via oral gavage daily, starting 1 day after subcutaneous injection of YUMM1.5 cells. Mice were treated with kynurenine, indoxyl sulfate, indole pyruvate, indole lactic acid, and indole (*n* = 6 mice/treatment). (D) Staining with anti-mouse CD8, CD11c, NK1.1, and F4/80 antibodies of tumor sections (control, *n* = 3; indoles, *n* = 3). CD8^+^, CD11c^+^, NK1.1^+^, and F4/80^+^ positive cells were quantified. For image analysis, 10 images were randomly selected from different areas of each tumor, in which the number of positive cells per section were counted. Scale bars, 50 μm. (E) Spatial distribution of immune cell types in tumor samples and their quantification (control, *n* = 3; indoles, *n* = 3). (F) Representative images of spatial distribution for niche enrichment (control, *n* = 3; indoles, *n* = 3). (G) Bar chart showing the composition of the cell types in different niches. Data shown represent two experiments. All data were analyzed by unpaired *t* test. Represented as mean ± SEM; ns, not significant; ∗∗*p* < 0.005, ∗∗∗∗*p* < 0.0001 using two-tailed *t* test or two-way ANOVA. See also Figure S5

To confirm that tryptophan degradation is required for the anti-tumor immunity promoted by *B. uniformis,* we deleted the *TnaA* gene by allelic exchange in the *B. uniformis* genome. PCR was carried out to confirm the deletion of *TnaA,* compared with the WT form of *B. uniformis* (Figure S5A). In agreement, indole production, which was monitored *in vitro* in cultures of the bacterial secretome of either the *TnaA* mutant or the WT forms of *B. uniformis*, revealed a significant decrease in the *TnaA* mutant compared with the WT *B. uniformis* strain (Figure S5B). Notably, while colonization with WT *B. uniformis* inhibited melanoma tumor growth in GF and ASF-bearing GF mice, this protective effect was absent in mice colonized with *TnaA*-deleted *B. uniformis*. Overall, these findings strongly suggest that tryptophan degradation by a single gut bacterial strain is required to inhibit melanoma growth (Figures 5A and 5B; Figures S5C and S5D). To further confirm the loss of indole production, we performed metabolomic analysis of indoles in the cecal samples from mice that were mono-colonized with either the WT or *TnaA*-deleted forms of *B. uniformis*. This analysis confirmed that mice colonized with WT but not the *TnaA* mutant form of *B. uniformis* produced indole (Figure S5E).

We next analyzed cytokine levels in the sera of GF mice colonized with ASF plus *B. rodentium* compared with ASF-only controls. That analysis revealed that GF administered with *B. rodentium* plus ASF showed lower levels of G-CSF and CXCL1 than did GF mice harboring only the ASF (Figure S5F). Similarly, G-CSF and CXCL1 levels were lower in the sera from both ASF-bearing GF and conventional C57BL/6J mice administered either *B. rodentium* or *B. uniformis* than in the sera from mice colonized with the *TnaA*-mutant *B. uniformis* (Figure S5F). Both G-CSF and CXCL1 (also known as keratinocyte chemoattractant [KC]) cytokines have been previously implicated in the inhibition of anti-tumor immunity.^30^^,^^31^^,^^32^^,^^33^ These findings suggest that G-CSF and CXCL1 inhibition by either *B. rodentium* or *B. uniformis* may serve to promote the activation of factors associated with immune activation against melanoma.

### Indoles are sufficient to inhibit melanoma growth in mice

We next asked whether tryptophan degradation products such as indoles could phenocopy the anti-tumor effects of *B. uniformis* or *B. rodentium*. To do so, we administered metabolites produced by either *TnaA* or *ArAT* enzymes, which are encoded by these bacterial strains, by daily oral gavage to conventional C57BL/6J mice. The degree of tumor inhibition was then compared with that of mice administered metabolites, which are produced by host enzymes upon tryptophan degradation and are linked to the IDO pathway. Notably, we found that metabolites such as indole and indoxyl sulfate (both *TnaA* breakdown products) as well as indole lactic acid and indole pyruvate (*AraT* breakdown products) effectively inhibited melanoma growth in these mice. Conversely, kynurenine, a metabolite produced by the host via the IDO pathway, did not inhibit tumor growth (Figure 5C and 5G).

We next determined whether the administration of indoles induces anti-tumor immunity as was observed following the administration of *B. rodentium* and *B. uniformis*. To this end, we performed RT-qPCR analyses of select genes in melanoma tumors from mice subjected to treatment with indoles, compared with controls. Notably, indoles promoted an increase in the expression of CD8, CD4, CD3, and NK1 (Figure S5H). Further analysis revealed an increase in the infiltration of CD8^+^ T cells, CD11c^+^ cells, and F4/80^+^ cells into tumors obtained from mice treated with indoles compared with controls (Figure 5D). Immunophenotyping using FACS, performed 12 days after melanoma tumor cell inoculation into conventional C57BL/6J mice, identified increased infiltration of CD44^+^, CD69^+^, and CD62L^+^ on CD8^+^ T cells from CD45-enriched cell populations. Increased expression of GZMB, TNFα, and IFNγ was noted on CD8^+^ and CD4^+^ T cells isolated from the tumors of mice treated with indoles compared with controls, in conventional C57BL/6J mice. These findings coincided with increased expression of the checkpoint receptor PD1 on CD8^+^ T cells (Figure S5I). Independent analyses were performed to determine the spatial distribution of immune cells within the tumor. Confirming earlier observations, we identified that tumors that were obtained from mice that were treated with indoles exhibited increased infiltration of T cells populations, among which the infiltration of CD8^+^ effector cytotoxic T cells and macrophage populations were most pronounced (Figure 5E). Evaluation of different niche populations revealed that tumors that were obtained from indole-treated mice exhibited increase in niches associated with the presence of CAFs, macrophages, and T cells (niches 2 and 6; Figures 5F and 5G). Collectively, these findings confirm that indoles promote anti-tumor immunity.

As indoles serve as ligands to activate the transcription factor AhR,^34^^,^^35^ we asked whether AhR signaling is involved in melanoma inhibition induced by indoles. To this end, we monitored the changes in the expression of AhR signaling components by RT-qPCR in tumors from mice subjected to indole treatment compared with controls. This analysis identified increased expression of CyP1B1 genes and decreased expression of TIPARP, BAFF, and CyP1A1 (Figures S6A and S6B). To further assess whether AhR signaling pathways may be involved in melanoma growth inhibition induced upon *B. rodentium* inoculation, we analyze the effect of the AhR agonist TCCB *in vivo*. While GF mice colonized with ASF plus *B. rodentium* inhibited melanoma growth, the addition of TCCB to this setting abolished this inhibition (Figure S6C). These data imply that AhR may not mediate tumor growth inhibition seen upon *B. rodentium* or indole administration. As indoles also can activate PXR and GPCR signaling pathways, we perform RT-qPCR of the components associated with these pathways. Indole administration did not increase the expression of the components associated with PXR (ABCB1, ZO-1, and NF-kB) or GPCR (FOS, EGR1, JUN, NR4a1, and IL-6; Figures S6D and S6E). These observations suggest that signaling pathways other than AhR, GPCR, and PXR may mediate indole’s ability to induce anti-tumor immunity, an important aspect that will be elucidated in future studies.

### *ArAT* and *TnaA* expression increase in patients who respond to ICB

**Figure 6:**
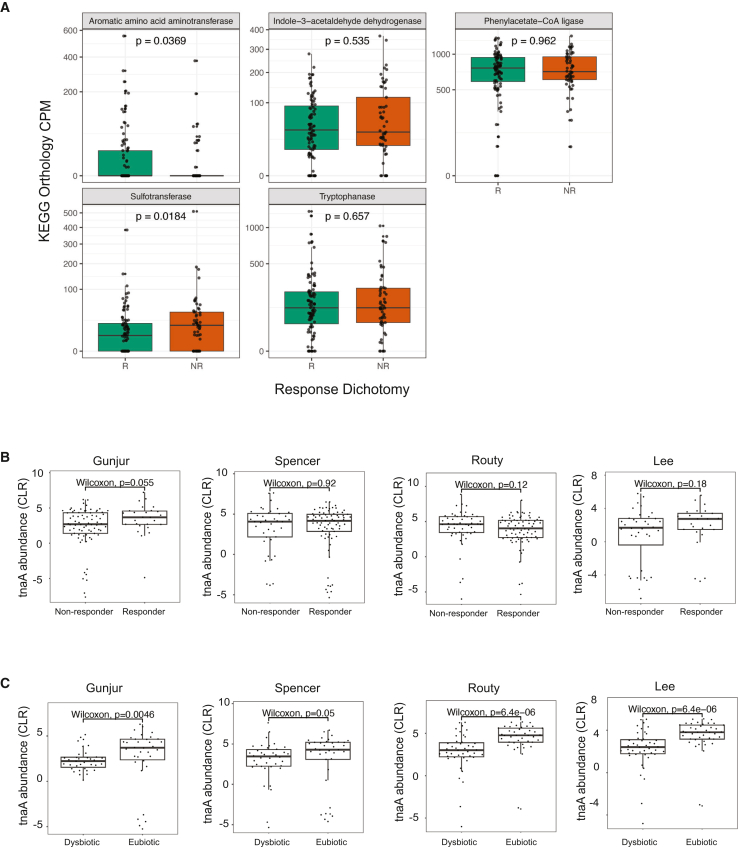
*ArAT* and *TnaA* expression increases in patients who respond to ICB (A) Tryptophan metabolism pathway activity by immunotherapy response in patients with melanoma. Imputed metabolomic pathway activity for selected Kyoto Encyclopedia of Genes and Genomes (KEGG) tryptophan metabolism enzymes^43^ derived from fecal metagenomic sequencing data. Cohort consists of patients with melanoma receiving immunotherapy as previously described.^36^ Each panel displays gene-level abundance (counts per million [CPM]) for the indicated KEGG orthology, stratified by response dichotomy (R, responders; NR, non-responders). Boxes represent the interquartile range (IQR) with the median line; whiskers extend to 1.5× IQR. Statistical comparisons performed using the Wilcoxon rank-sum test; *p* values are shown above each comparison. (B) Abundance of *TnaA* in Ganjur,^36^ Spencer,^43^ Routy,^44^ and Lee^37^ cohorts, comparing responders vs. non-responders to immune checkpoint therapy. (C) Abundance of *TnaA* in Ganjur,^36^ Spencer,^43^ Routy,^44^ and Lee^37^ cohorts. Functional dysbiosis was computed for all the samples (described in Liu et al.^45^). Samples were classified, per study, as dysbiotic (top tertile of functional dysbiosis) or eubiotic (bottom tertile of functional dysbiosis) levels.

Given that immune checkpoint therapy is currently the first-line therapy for patients with melanoma, we analyzed stool samples from different human cohorts of patients with melanoma, comparing responders versus non-responders to ICB therapy, to identify possible differences in the level of enzymes implicated in tryptophan degradation. This analysis identified a significant increase in the level of *ArAT* in patients who respond to ICB therapies in one of the cohorts (Figure 6A) and an increase in *TnaA* levels in Gunjur^36^ and Lee^37^ cohorts (Figure 6B). In addition, we analyzed dysbiosis levels in these cohorts, and the *TnaA* levels were found to be elevated in patients who are eubiotic compared with those who have dysbiosis (Figure 6C). In agreement, increase in metabolites or enzymes implicated in tryptophan degradation pathways was independently observed in patients who responded to ICB therapies in different cancer types,^38^ such as melanoma,^39^ non-small cell lung cancer,^40^^,^^41^ and CRC.^42^ These findings establish the human relevance of our finding that tryptophan degradation is implicated in the inhibition of melanoma and identifies indoles, produced by either *B. rodentium* or *B. uniformis,* as important metabolites sufficient to mediate the inhibition of melanoma growth.

## Discussion

Here, we identify select bacterial species and their respective metabolites as capable of limiting melanoma growth. Both the mouse and human strains (*B. rodentium* and *B. uniformis*, respectively) were shown to be capable of inhibiting melanoma, as well as other tumors, in different cancer models and mouse strains. This inhibition required the bacterial enzymes *TnaA* and *ArAT*, which are important in tryptophan breakdown to indoles. While mutation of *TnaA* in *B. uniformis* was sufficient to abolish the tumor inhibition phenotype, elevated levels of *ArAT* was found in patients who responded to ICB.

While our initial findings were based on colonization of select bacteria in GF mice, subsequent experiments were performed using mouse models with more complex microbiomes, including GF mice colonized with a microbiota devoid of *Bacteroides* species and conventional C57BL/6J mice, all of which showed anti-tumor effects resembling those seen in GF mice. We found that changes elicited by tryptophan breakdown were linked to activation of intestinal immune cells, inhibition of serum factors thought to limit anti-tumor immunity (CXCL1 and G-CSF), and tumor infiltrations by CD8^+^ T cells, activities coinciding with enhanced anti-tumor immunity.

The importance of the gut microbiota in controlling anti-tumor immunity and tumor growth has been intensively investigated in several tumor models.^3^^,^^46^^,^^47^^,^^48^^,^^49^ In mouse models of pancreatic cancer, indoles produced in the gut by *Lactobacillus* species reduced anti-tumor immunity by activating the AhR in tumor cells and thereby decreasing CD8^+^ T cell levels and increasing tumor growth.^19^ In contrast, the metabolite indole-3-aldehyde (I3A) produced by *Limosilactobacillus reuteri* increased chemotherapy efficacy in mouse models of pancreatic cancer, and patients with pancreatic cancer who responded to therapy showed enrichment of *Lactobacillus reuteri*^50^ in the gut. I3A has also been linked to enhanced immune checkpoint inhibitor efficacy against melanoma in mouse models and in patients.^39^ Conversely, indole-3-lactic acid, which is produced by *Lactobacillus gallinarum*, has been shown to limit CRC by inhibiting AhR expression in tumor cells.^51^ While a number of AhR ligands were shown to impact the degree of anti-tumor immunity, future studies will clarify which of the cellular signaling components mediate the ability of *B. rodentium*/*B. uniformis* or indole to induce anti-tumor immunity.

Importantly, we observed that *B. uniformis* colonization also inhibited melanoma growth, phenocopying the effects of *B. rodentium*. Both of these species harbor genes encoding the tryptophan-degrading enzymes *TnaA* and *ArAT*, which produce different types of indoles as degradation products. Notably, each of the indoles tested here for anti-tumor activity, whether produced by *TnaA* or *ArAT*, was sufficient to inhibit melanoma growth. These findings coincide with human data showing that elevated *ArAT* expression is seen in patients with melanoma who respond to ICB. Along these lines, the presence of *B. uniformis* has been linked with remission of patients with cancer.^52^^,^^53^ Along these lines, metataxonomic microbiome analyses of responders to PD1 therapy also pointed to a positive correlation between treatment responsiveness and *B. uniformis* abundance.^44^ Overall, our findings suggest that indoles produced by the enzymes present in *B. uniformis* are critical for tumor growth inhibition, an effect shown here for melanoma, breast, colon, and pancreatic cancers, indicative of a general phenomenon across different genetic mouse models.

Independent studies suggest that the mechanisms underpinning *B. uniformis* anti-tumor activity may involve the gut-brain axis. In rats, colonization of *B. uniformis* CECT 7771 has been reported to impact brain circuitry functioning in the reward response.^54^ Given that the brain reward system is implicated in enhancing anti-tumor immunity and limiting tumor growth in mice,^55^ the activity of the gut-brain axis^56^ may also underlie the effects of *B. uniformis* in our model. *B. uniformis* has also been implicated in attenuating intestinal inflammation as its administration restored intestinal barrier function, increased NF-κB activity and mitogen-activated protein kinase (MAPK) signaling in colonic tissues, and promoted TH17 cell differentiation in a mouse model of chemically induced colitis.^57^ Such findings suggest that enhancing anti-tumor immunity via *B. uniformis* may also impact auto-immune responses, which often emerge in response to immune checkpoint therapies,^58^^,^^59^ exemplified in the *Rnf5* KO mice, which exhibit both enhanced anti-tumor immunity and auto-immunity.^4^^,^^11^

In all, this study highlights an approach for eliciting effective anti-tumor immunity to inhibit tumor growth, an activity that requires indole production due to degradation of tryptophan by *Bacteroides* species in the mouse and human intestines. The finding that indoles serve as key mediators of anti-tumor phenotypes may offer alternative paths for clinical evaluation of the tryptophan degradation pathway, distinct from previously attempted approaches to inhibit IDO1. Our findings provide the foundation for treatment modalities that could complement or replace existing ones.

### Limitations of the study

While our data suggest that the canonical transcriptional signatures of AhR, GPCR, or PXR activation are not strongly induced following *B. rodentium* or indole administration, further work is needed to define the mechanisms underlying indoles’ ability to induce ATI. It is important to note that a partial, context-dependent, or cell type-specific AhR/GPCR/PXR signaling cannot be excluded. Based on our finding, it will be also important to determine whether enhanced administration of *ArAT* or *TnaA*, or their combination, could offer an effective means to induce ATI, a subject tor future studies.

## Resource availability

### Lead contact

Requests for further information and resources should be directed to and will be fulfilled by the lead contact, Ze’ev A. Ronai (zeev.ronai@csmc.edu).

### Materials availability

Reagents generated in this study will be made available on request, but we may require payment and/or a completed materials transfer agreement if there is potential for commercial application. There are restrictions to the availability of *TnaA*-mutant *B. uniformis* because of the lack of an external centralized repository for its distribution and our need to maintain the stock. We are glad to share this mutant bacterial strain with reasonable compensation by requestor for its processing and shipping.

### Data and code availability

Data

• RNA sequencing data have been deposited at GEO under accession number GEO: GSE293242. 16S sequencing data have been deposited at NCBI SRA under BioProject ID: PRJNA1474073. Metabolomics data have been deposited at Metabolomic Workbench study ST004939 (project PR003168, doi: https://doi.org/10.21228/M86273). These data are publicly available as of the date of publication. Microscopy data reported in this paper will be shared by the lead contact upon request.
• This paper analyzes existing, publicly available data, accessible in the references Gunjur et al.,^36^ Lee et al.,^37^ Liu et al.,^45^ Dohlman et al.,^60^ and Mimpen et al.^61^ for the analyses performed by NKI collaborators and accessible in the references Spencer et al.^43^ and Gopalakrishnan et al.^62^ for the analyses performed by MDACC.

Code.

• This paper does not report original code.
Additional information
• Any additional information required to reanalyze the data reported in this paper is available from the lead contact upon request.

## Acknowledgments

We thank members of the Ronai lab for extensive discussions. We thank Dr. Cosimo Commisso of Sanford Burnham Medical Discovery Institute, for providing us with the KP65 cells. We also thank Lorin Chin of the Cedars Sinai Microbiome Institute as well as Mason Mandolfo and Robert Schmaltz of the University of Nebraska-Lincoln for their assistance with bacterial cultures and animal studies and Dina Abbasian of the University of Pennsylvania for metabolic analyses. Support by the Cedars-Sinai shared resources in genomics, vivarium, and microbiome studies is greatly appreciated. We gratefully acknowledge support by NCI grant R35CA197465 (to Z.A.R.), by gift from the Hervey Family/San Diego Foundation (to Z.A.R.), by grant R21CA249822 (to A.E.R.-T. and H.K.), and by funding from the Buffett Cancer Center funds (to A.E.R.-T.) via NCI grant CA036727.

## Author contributions

X.D.O., A.E.R.-T., and Z.A.R. conceived the study; X.D.O., K.B., and G.P. performed the experiments; D.S. and C.P. performed metabolite analyses; A.K.S. and A.M. analyzed bacterial 16S rRNA gene sequencing data; S.K. oversaw spa tial analysis, T.W.Z. and A.S. performed bioinformatic analyses; N.J.A., J.W., E.E.V., and M.P.M. analyzed indicated patient stool samples. E.M., S.D., A.O., M.B.F., O.H., A.E.R.-T., N.J.A., J.W., and Z.A.R analyzed the data; X.D.O., A.E.R.-T., and Z.A.R. wrote the manuscript with contributions from all authors.

## Declaration of interests

A provisional patent application based on the findings disclosed in this manuscript was submitted.

## Declaration of generative AI and AI-assisted technologies in the writing process

No generative or AI-assisted technologies were used in the writing process.

## STAR★Methods

### Key resources table

**Table:** 

REAGENT or RESOURCE	SOURCE	IDENTIFIER
**Antibodies**		

B220 (Clone: RA3-6B2)	Biolegend	Cat#103222: RRID:AB_313005
CD11b (Clone: M1/70)	Biolegend	Cat#101224: RRID:AB_755986
CD11c (Clone: N418)	Biolegend	Cat#117310: RRID:AB_313779
CD16/32 (Clone: 93)	Biolegend	Cat#101302: RRID:AB_312801
CD206 (Clone: C068C2)	Biolegend	Cat#141721: RRID:AB_2562340
CD25 (Clone: 3C7)	Biolegend	Cat#101907: RRID:AB_961210
CD279 (Clone: 29F.1A12)	Biolegend	Cat#135210: RRID:AB_2159183
CD4 (Clone: RM4-5)	Biolegend	Cat#100548: RRID:AB_2563054
CD40 (Clone: HM40-3)	Biolegend	Cat#102906: RRID:AB_312949
CD44 (Clone: IM7)	Biolegend	Cat#103028: RRID:AB_830785
CD45 (Clone: 104)	Biolegend	Cat#109822: RRID:AB_493731
CD69 (Clone: H1.2F3)	Biolegend	Cat#104511:RRID:AB_493565
CD8 (Clone: 53–6.7)	Biolegend	Cat#100725: RRID:AB_493425
CD80 (Clone: 16-10A1)	Biolegend	Cat#104733: RRID:AB_2563112
F4/80 (Clone: BM8)	Biolegend	Cat#123108: RRID:AB_89350
GR1 (Clone: RB6-8C5)	Biolegend	Cat#108407: RRID:AB_313372
Lag3 (Clone: C9B7W)	Biolegend	Cat#125208: RRID:AB_2133343
MHC I (Clone: AF6-88.5)	Biolegend	Cat#116515: RRID:AB_1967107
MHC II (Clone: M5/114.15.2)	Biolegend	Cat#107623: RRID:AB_893586
NK1.1 (Clone: OK136)	Biolegend	Cat#108728: RRID:AB_2132705
PDCA-1 (Clone: 129c1)	Biolegend	Cat#127104: RRID:AB_1953283
CD8α (D4W2Z) Rabbit mAb	Cell signaling	Cat#98941: RRID:AB_275637
CD11c (D1V9Y) Rabbit mAb	Cell signaling	Cat#97585: RRID:AB_2800282
Alexa Fluor 594-conjugated, Donkey, anti-rabbit IgG	Invitrogen	Cat# A21207
InVivoMAb rat IgG2a isotype control, anti-trinitropheno	BioXcell	Cat# BE0089
InVivoMAb anti-mouse PD-1 (CD279)	BioXcell	Cat# BE0146
Epacadostat	MedChemExpress	Cat# HY-15689

**Bacterial and virus strains**		

ASF 356, *Clostridium* sp	Cecal Mice UNL	N/A
ASF 360, *Lactobacillus intestinalis*	Cecal Mice UNL	N/A
ASF 361, *Lactobacillus murinus*	Cecal Mice UNL	N/A
ASF 457, *Mucispirillum schaedleri*	Cecal Mice UNL	N/A
ASF 492, *Eubacterium plexicaudatum*	Cecal Mice UNL	N/A
ASF 500, *Pseudoflavonifractor* sp	Cecal Mice UNL	N/A
ASF 502, *Clostridium* sp	Cecal Mice UNL	N/A
ASF 519, *Parabacteroides goldsteinii*	Cecal Mice UNL	N/A
*Bacteroides rodentium*	DSMZ	Cat#26882
*Bacteroides uniformis*	ATCC	Cat#8492
*Bacteroides uniformis mutated*	University of Michigan	N/A
*Phocaeicola massiliensis*	DSMZ	Cat#17679
*Phocaeicola sartorii*	DSMZ	Cat#21941

**Chemicals, peptides, and recombinant proteins**		

10,000 I.U/mL penicillin, 10mg/mL streptomycin, 200mM L-glutamine (100X PSG)	Cytiva	Cat# SV30010
Antibody Diluent, Background Reducing	Agilent	Cat#S302281-2
CAS-Block TM	Invitrogen	Cat#8120
Collagenase D	Sigma Aldrich	Cat# COLLD-RO
Deoxyribonuclease I from bovine pancreas	Sigma Aldrich	Cat# DN25
Dimethyl Sulfoxide	Sigma Aldrich	Cat# D2650
DPBS (1X)	Thermo	Cat#14190-250
Epitophe Retrieval Solution, pH 6	Leica	Cat# RE7113-CE
Ethanol, 190 proof (95%), USP, Decon™ Labs	Fisher Scientific	Cat#04-355-722
Fetal Bovine Serum (FBS)	Sera Prime	Cat# F31016
Formaldehyde, 37 wt % sol. in water, stab. with 5–15% methanol	Thermo Fisher Scientific	Cat#119690010
Gibco™ Trypsin-EDTA (0.05%), phenol red	Fisher Scientific	Cat#25-300-120
Invitrogen™ RNA*later*™ Stabilization Solution	Fisher Scientific	Cat# AM7020
Kynurenic acid	Sigma Aldrich	Cat# K3375
Recombinant Mouse GM-CSF (carrier-free)	Biolegend	Cat#576304
SsoAdvanced Universal SYBR Green Supermix	BIO-RAD	Cat#1725274
Taurine	Sigma Aldrich	Cat# T0625
UltraPure TM Distilled Water DNase, RNase, Free	Invitrogen	Cat#10977-015
VECTASHIELD® Antifade Mounting Medium with DAPI (H-1200-10)	VECTASHIELD	Cat# H-1200-10
Xylenes	Fisher Chemical	Cat# C8H10
Z-FIX ZINC FIXATIVE	Fisher Scientific	Cat# NC9378601
L-Kynurenine	Milipore sigma	Cat# K8625
Indoxyl sulfate potassium salt	Milipore sigma	Cat# I3875
Indole-3-pyruvic acid	Milipore sigma	Cat# I7017
Indole	Milipore sigma	Cat# I3408
DL-Indole-3-Lactic acid	Milipore sigma	Cat# I5508

**Critical commercial assays**		

Bio-Rad Quote for Bio-Plex Pro Mouse Cytokine 23-plex Assay	Bio Rad	N/A
High-Capacity cDNA Reverse Transcription Kit only	Thermo	Cat#4368814
Monarch® Total RNA Miniprep Kit	New England Biolabs	Cat#T2010S
QIAamp Fast DNA stool Mini kit (50)	Qiagen	Cat#51604

**Deposited data**		

Metabolomic	This paper	Metabolomic Workbench ST004939
16S sequencing	This paper	NCBI SRA, BioProject ID: PRJNA1474073
RNA-seq	This paper	GEO: GSE 293242

**Experimental models: Cell lines**		

YUMM1.5	This paper	N/A
SW1	This paper	N/A
MC38	This paper	N/A
4T1	This paper	N/A

**Experimental models: Organisms/strains**		

GF C57BL/6J	University of Nebraska-Lincoln	N/A
C57BL/6J	Jackson Laboratories	Cat#000664, RRID:IMSR_JAX:000664
C3H	Charles River Laboratories	Cat#025, RRID:IMSR_CRL:025
BALB/cj	Jackson Laboratories	Cat# 000651, RRID:IMSR_JAX:000651

**Oligonucleotides**		

CD86 Forward (5′-3′) TGTTTCCGTGGAGACGCAAG	This paper	N/A
CD86 Reverse (5′-3′) TTGAGCCTTTGTAAATGGGCA	This paper	N/A
ICAM Forward (5′-3′) GTGATGCTCAGGTATCCATCCA	This paper	N/A
ICAM Reverse (5′-3′) CACAGTTCTCAAAGCACAGCG	This paper	N/A
CCR1 Forward (5′-3′) CTCATGCAGCATAGGAGGCTT	This paper	N/A
CCR1 Reverse (5′-3′) ACATGGCATCACCAAAAATCCA	This paper	N/A
TLR1 Forward (5′-3′) TGAGGGTCCTGATAATGTCCTAC	This paper	N/A
TLR1 Reverse (5′-3′) AGAGGTCCAAATGCTTGAGGC	This paper	N/A
TLR2 Forward (5′-3′) GCAAACGCTGTTCTGCTCAG	This paper	N/A
TLR2 Reverse (5′-3′) AGGCGTCTCCCTCTATTGTATT	This paper	N/A

**Software and algorithms**		

FACS FlowJo v10 Analyser	BD Biosciences	N/A
GraphPad Prism 10	GraphPad	https://www.graphpad.com/features
ImageJ	–	https://imagej.net/ij/
Adobe Illustrator	Adobe	https://www.adobe.com/products/illustrator.html
Thermo Chromelon Software	Thermo Fisher Scientific	N/A
Pathway Analysis (IPA)	Qiagen	N/A
Luminex xPONENT software	Thermo Fisher Scientific	N/A

**Other**		

Amino Acid diet	Envigo	Cat# TD.01084
Tryptophan Deficient AA Diet	Envigo	Cat# TD.180506
JL Rat and Mouse/Auto 6F	LabDiet	Cat#5K67

### Experimental model and study participant details

#### Animals and tumor models

All experimental animal procedures were approved by the Institutional Animal Care and Use Committee of Sanford Burnham Prebys Medical Discovery Institute (SBP: approval #22–080) and Cedars-Sinai Medical Center (Protocol: IPROTO202400000337) and complied with respective ethical animal testing and research regulations. Male mice, 6-8 weeks-old, were used for all experiments. Male mice were used since the melanoma cell lines were generated in male mice. Conventional C57BL/6J mice, C3H, and Balb/c mice were purchased from Jackson Laboratories. GF C57BL/6J mice were bred and maintained at the University of Nebraska-Lincoln (UNL) by the Nebraska Gnotobiotic Mouse Program under gnotobiotic conditions in flexible film isolators. The Institutional Animal Care and Use Committee at UNL approved experiments involving GF mice (protocols #2126 and #2622). All GF mice were fed autoclaved chow diet *ad libitum* (LabDiet 5K67, Purina Foods). GF status of the breeding colony was routinely checked as described.^63^ Mice were injected subcutaneously (s.c.) with either 1x10^6^ YUMM1.5 melanoma cells, 8x10^5^ SW1, melanoma cells, 5x10^5^ 4T1 breast tumor cells, 2x10^5^ KP65, pancreatic cells or 5x10^5^ MC38, colorectal cancer cells. Tumor size was measured twice a week to calculate tumor volume. Tumors were weighed at the time of excision.

#### Cell lines

This study used the melanoma line YUMM1.5^64^ and SW1,^65^ colorectal line MC38, pancreatic cell line KP65 (kindly provided by Dr. C. Commisso) and breast cancer cell line 4T1, cultured in Dulbecco’s modified Eagle’s medium supplemented with 10% fetal bovine serum (FBS; Sera prime, Cat# F31016) and antibiotics. Cells were authenticated at the Cedars-Sinai Medical Center facility and continuously monitored for mycoplasma-free status.

#### Analysis of clinical databases

For microbiome analysis, we utilized microbiome abundance data from TCMA (The Cancer Microbiome Atlas).^60^ The dataset comprises microbiome abundance profiles from 620 solid tumors in TCGA studies (including HNSC, ESA, STAD, COAD, READ). We extracted relative abundance data at both the genus level (*Bacteroides*) and the species level (*Bacteroides uniformis*). For survival analysis, clinical data were updated based on harmonized outcomes reported by Liu et al.^45^ with endpoint definitions following their recommendations.

Kaplan-Meier survival curves were generated separately for the taxon and endpoints shown in each panel, with survival time plotted in years and p-values determined by two-sided log rank tests. For Cox proportional hazards models, survival time was analyzed in days. The reported hazard ratios compare Present versus Absent bacterial state and were adjusted for AJCC tumor stage, age at initial pathologic diagnosis, and sex; tumor type was evaluated as an additional sensitivity covariate where applicable. Patients with missing endpoint data were excluded from the corresponding Kaplan-Meier analysis, and patients with unreported tumor stage were excluded from adjusted Cox models.

Bacterial abundance was dichotomized before survival modeling: relative abundance greater than 0 was classified as Present, and abundance equal to 0 was classified as Absent. For the genus-level Bacteroides overall survival analysis shown in Figure S1E, TCMA genus-level relative-abundance values were collapsed to the patient level by trimming aliquot barcodes to the first 12 TCGA characters and retaining the sample with the highest abundance when multiple aliquots mapped to the same patient. After merging with Liu et al. clinical outcomes, 503 patients had overall-survival endpoint data (Absent, *n* = 337 with 136 deaths; Present, *n* = 166 with 35 deaths), and 454 patients with reported tumor stage were included in the adjusted Cox model (153 deaths). This analysis included HNSC, STAD, COAD, ESCA, and READ cases.

For the species-level *B. uniformis* disease-free interval analysis shown in Figure 4D, TCMA whole-exome sequencing solid-tumor case species-level relative-abundance values were filtered to NCBI taxon ID 820 (*Bacteroides uniformis*) and merged by TCGA patient barcode. After merging with Liu et al. clinical outcomes, 224 patients had disease-free interval endpoint data (Absent, *n* = 124 with 21 events; Present, *n* = 100 with 9 events), and 213 patients with reported tumor stage were included in the adjusted Cox model (28 events). This species-level analysis included COAD and READ cases.

The analysis of the abundance of *TnaA* between responders vs. non-responders and based on functional dysbiosis levels was performed as outlined before.^45^ Briefly, data was processed using Metaphlan V4 and human V3 to obtain quantifications of Kegg Orthologs present in the microbial community. For *TnaA* quantifications, the centered log ratio abundance of the Kegg Ortholog K01667 were used. Pre-treatment samples were analyzed from the following datasets: Lee: PRJEB43119, using the PRIMM cohorts,^61^ Gunjur: PRJEB49516,^36^ Spencer: PRJNA770295,^43^ and Routy: PRJEB22863.^44^

The patients analyzed in these cohorts were part of studies published before. In all cases the patient population consists of both females and males, aged 30-80 years old, from patient populations in southeastern Australia and Houston, Texas, USA. The race of the patients was mixed, as indicated in the corresponding publications.^36^^,^^43^^,^^44^^,^^45^^,^^61^

### Method details

#### Culture and administration of bacterial strains

Two microbiomes—the Altered Schaedler Flora (ASF) and MC608-F-a1—were used to inoculate GF mice. The ASF consists of eight strains: ASF 356, *Clostridium* sp.; ASF 360, *Lactobacillus intestinalis*; ASF 361, *Lactobacillus murinus*; ASF 457, *Mucispirillum schaedleri*; ASF 492, *Eubacterium plexicaudatum*; ASF 500, *Pseudoflavonifractor* sp.; ASF 502, *Clostridium* sp.; and ASF 519, *Parabacteroides goldsteinii*. MC608-F-a1 refers to mice derived from a wild mouse population in the Massif Central region of France previously maintained at the Max Planck Institute for Evolutionary Biology (Plon, Germany; referred to as MC608-F-a1 in^22^^,^^23^ and as A in^24^). Ceca were collected from either ASF- or MC608-F-a1-bearing mice, stored at −80°C, and the contents resuspended (1:3 wt/vol) in reduced PBS as previously described^63^ at the time of use.

Two weeks before tumor cell injection, 100 μL of cecal contents (either from ASF- or MC608-Fa1-bearing mice) and bacterial strains of interest (1×10^6^ to 1×10^7^) were administered to GF mice via three oral gavages one day apart. Bacterial strains used in this study included: *Bacteroides rodentium* ST28 (DSMZ 26882), *Bacteroides uniformis* VPI 0061 (ATCC 8492), *Phocaeicola massiliensis* B84634 (DSM 17679), and *Phocaeicola sartorii* AC20 (DSM 21941). For experiments performed in conventional C57BL/6J mice, *B. rodentium* and *B. uniformis* were colonized via oral gavage three times a week for the duration of the study.

#### Deletion of the *B. uniformis tnaA* gene by allelic exchange

A pLGB13-based^66^^,^^67^ gene deletion plasmid (pLGB13-ΔtnaA) for deleting tnaA was constructed using Phusion High-Fidelity DNA Polymerase for all PCR steps. Briefly, the upstream and downstream regions (∼1500bp) of the *tnaA* gene were amplified from the *Bacteroides uniformis* genome using the following two primer sets: (upstream: TAAATCTCTTACATAGACTCTGTATCTTTAATAATTTGACA and ACTGGAAGATAGGCAATTAGGAAGAAGAGCAAGTACGGCTG) and (downstream: GTAAGATTAGCATTATGAGTGGTTCCACCTCTTTCTTGAACAAGC and CAGAGTCTATGTAAGAGATTTACAGACTCAATGTCA), respectively. PCR products were visualized on a 1% agarose gel, and fragments of the expected size were gel purified using the QIAquick Gel Extraction Kit (Qiagen). The deletion vector pLGB13 was linearized by double digestion with BamHI and SalI restriction enzymes (New England Biolabs). Digested pLGB13 and the gel-purified upstream and downstream *tnaA* fragments were then ligated using a Gibson Assembly Master Mix (New England Biolabs), following the manufacturer’s protocol. The assembled plasmid was transformed into electrocompetent *E. coli* strain S17-λ*pir* and transformants were selected on LB agar plates supplemented with 200 μg/mL ampicillin. Successful insertion of the upstream and downstream regions was confirmed by PCR using primers that lie inside the 1.5 kb fragment used to create pLGB13-ΔtnaA (GTTTTCGTTGTTCACGATGGTT/and CTTGTCGGTCAGCTCCAAC). The entire pLGB13-ΔtnaA construct was then sequenced by Plasmidsaurus to verify the absence of off-target mutations and confirm the correct arrangement of upstream and downstream region.

The pLGB13-ΔtnaA plasmid in conjugative *E. coli* donor strain S17-λpir was used to construct the gene deletion. For conjugation, both the donor *E. coli* S17-λpir and the recipient *B. uniformis* were grown overnight. *E. coli* was cultured in LB broth supplemented with 200 μg/mL ampicillin at 37°C with shaking, while *B. uniformis* was cultured in TYG broth at 37°C under anaerobic conditions. On the day of conjugation, overnight cultures were subcultured (1:500 for *E.coli* and 1:100 for *B. uniformis*) into fresh respective media. Cells were incubated until they reached OD_600_ of approximately 0.1–0.2. Donor and recipient cultures were pelleted by centrifugation at 20,000 × g for 3 min and washed twice with sterile TYG to remove residual antibiotics. The donor and recipient cells were then resuspended in 200 μL of fresh TYG. Four 50 μL spots of the mixed bacterial suspension were then spotted directly onto pre-warmed Blood BHI agar plates and incubated aerobically at 37°C overnight. Following incubation, bacteria from the mating spots were resuspended in 1 mL of sterile TYG. Serial dilutions of this suspension were then plated onto selective Blood BHI agar plates containing 25 μg/mL erythromycin and 200 μg/mL gentamicin. Plates were incubated anaerobically at 37°C for 2 to 3 days. Individual colonies (*n* = 12) were re-streaked for isolation on fresh selective plates to ensure purity.

Twelve isolated merodiploids (erythromycin resistant colonies containing the *tnaA* deletion plasmid integrated via one of the cloned gene-flanking fragments) were individually grown overnight in 5 mL of TYG broth. These cultures were then mixed in equal volumes, serially diluted, and plated onto BHI agar blood agar plates containing 100 ng/mL anhydrotetracycline (aTC). Plates were incubated anaerobically at 37°C for 2 to 4 days. Single colonies that grew on aTC-containing plates were re-streaked for isolation. Loss of the target *tnaA* gene was confirmed by genomic PCR using the upstream and downstream nested primers GTTTTCGTTGTTCACGATGGTT and CTTGTCGGTCAGCTCCAAC, respectively, and testing in medium containing erythromycin to confirm that the plasmid was also lost from the strain (*i.e*., clones had regained sensitivity to this antibiotic).

#### Dietary manipulations

For dietary experiments, we used irradiated chow, either Amino Acid diet (TD.01084, Envigo) or Tryptophan Deficient AA Diet (TD.180506, Envigo). Diets were administered to mice starting 3 days before tumor inoculation until the end of the experiment.

#### Taurine, kynurenic acid, kynurenine, and indoles administration

Taurine (10 mg/mL, Sigma Aldrich) was administered in drinking water 3 weeks before tumor cell injection until the end of the experiment. Sterile water served as vehicle control. Kynurenic acid (Sigma Aldrich; 40 mg/kg body weight) in 200 μL sterile water was administered daily by oral gavage starting 3 days before tumor cell inoculation until the end of the experiment. Kynurenine, indoxyl sulfate, indole pyruvate, indole 3 lactic acid and indole (All from Sigma Aldrich; 40mg/kg body weight) in 100 mL of corn oil was administered daily by oral gavage starting 1 day after tumor cell inoculation until the end of the experiment.

#### *In vivo* treatment

TCDD (Cambridge Isotope Laboratories), was administrated (oral gavage 5μg/kg) weekly, starting two weeks prior tumor injection.

#### RNA extraction and RT-qPCR analyses

Total RNA was extracted from mouse tumor samples using the Total RNA Miniprep Kit (Monarch). RNA purity and concentration was quantified by reading at 260 and 280 nm in a NanoDrop spectrophotometer (Thermo Fisher). RNA was reverse transcribed using a High-Capacity Reverse Transcriptase Kit (Invitrogen) according to the manufacturer’s protocol. RT-qPCR analysis was performed using SsoAdvanced Universal SYBR Green Supermix (Bio-Rad) and a Bio-Rad CFX Connect Real-Time system. Expression levels were normalized to H3N controls. The sequences of the primers used are listed in Table S1.

#### Gas chromatography-mass spectrometry (GC-MS) sample preparation and analysis

Samples of cecum contents (40–60 mg) or serum (15 μL) and standards were prepared as described,^68^ with additional standards as listed in Table S2. Samples and standards were analyzed by GC-MS using an TG-SQC column (15 m × 0.25 mm × 0.25 μm, Thermo) installed in a Thermo Scientific TSQ 9610 GC-MS/MS. The GC was programmed with an injection temperature of 300°C and a 0.5 μL injection. The GC oven temperature was initially 130°C for 4 min, rising to 250°C at a rate of 6°C/min, and to 310°C (280°C for serum) at 60°C/min with a hold at the final temperature for 2 min. GC flow rate with helium carrier gas was 50 cm/s. The GC-MS interface temperature was 300°C and (electron impact) ion source temperature was 200°C, with 70 eV ionization voltage. Standards were run in parallel with samples. Metabolites in samples and standards were detected by MS/MS using precursor and product ion masses, and collision energies shown in the attached table. Sample metabolites were quantified using calibration curves made from the standards in Themo Chromeleon software, and further data processing to adjust for the relative quantities of metabolites in the standards, and for recovery of the internal standard were done in MS Excel.

Independent metabolomic analysis was performed at Metware Biotechnology Inc. Samples stored at −80°C were thawed on ice. 400uL solution (Methanol: water = 7.3, V/V) containing internal standard were mixed with 20mg of the samples (vortexed for 3 min). Samples were than sonicated (ice bath for 10 min) and vortexed (1 min) followed by incubation (−20°C) for 30min. The samples were centrifugated (12000 rpm for 10 min) and the supernatant was subject for another centrifugation (12000rpm for 3min). 200uL of the supernatant was then used for LC-MS analysis. For untargeted detection, Ultra Performance Liquid Chromatography (UPLC) and Quadrupole-Time of Flight Spectrometry with a chromatographic column, ACQUITY HSS T3 (2.1 × 100mm, 1.8 μm) were used using ultrapure water (0.1% formic acid added), and acetonitrile (0.1% formic acid added) with column (temperature 40°C, flow rate 0.4mL/min, and injection volume of 5 μL). For widely targeted detection, UPLC and tandem mass spectrometry (MS/MS) (QTRAPr6500+) were employed using chromatographic column (Waters ACQUITY UPLC HSS T3 C18 1.8um, 2.1mm∗100mm). A phase was ultrapure water (0.1% formic acid added), B phase was acetonitrile (0.1% formic acid added); Gradient program was 95:5 V/V at 0 min, 10:90 V/V at 10,0min, 10:90 V/V at 11.0min, 95:5 V/V at 11.1min, 95:5 V/V at 14.0min; Flow rate was 0.4mL/min at 40°C, injection volume 2uL.

#### RNAseq analysis

RNA samples were prepared from mouse tumor samples from ASF alone and ASF plus *B. rodentium* samples collected at experiment’s end (Day 22). Samples contained 250 ng of RNA. For library construction, PolyA RNA isolation and library preparation were performed with the Watchmaker Genomics mRNA Library Prep Kit (Watchmaker Genomics) and Elevate Long UDI Adapters (Element Biosciences). RNA libraries were pooled and sequenced (2x75bp) on the Element Biosciences AVITI sequencer with the 2x75 Cloudbreak kit.

Illumina Truseq adapters and polyA/polyT sequences were trimmed from raw reads using Cutadapt v2.3,^69^ and trimmed reads were aligned to mouse genome version mm10 and Ensembl gene annotations v84 using STAR version 2.7.0days_0221,^70^ adopting alignment parameters from the ENCODE long RNA-seq pipeline (https://github.com/ENCODE-DCC/long-rna-seq-pipeline). RSEMv1.3.1 was used to obtain gene level estimated counts and transcripts per million.^71^

FastQC v0.11.5 (https://www.bioinformatics.babraham.ac.uk/projects/fastqc/) and MultiQC v1.8^72^ were used to assess quality of trimmed raw reads and alignment to the genome and transcriptome. Only genes with RSEM estimated counts ≥5 times the total number of samples were retained for differential expression analysis. Differential expression comparisons were performed using the Waldtest implemented in DESeq2 v1.22.2.^73^ Genes with a Benjamini– Hochberg-corrected *p*-value of< 0.05 and fold-change of ≥1.5 or ≤1.5 were defined as differentially expressed. Gene set enrichment analysis (GSEA) for TCGA-PRAD comparison of ASF versus ASF plus *B. rodentium* was performed using the pre-ranked option in GSEA version 4.2.3.^74^ GSEA for RNA-seq comparisons was performed using GSEA version 4.3.2, and RPKM values with the parameter “Permutation type = gene_set” Pathway analysis was performed using Ingenuity Pathway Analysis (IPA) software (Qiagen).

#### Histology and immunofluorescence

Tumor and small intestine samples were collected immediately after animals were sacrificed. The small intestine was split open lengthwise, rinsed, and rolled up from the proximal to distal end to form a “Swiss roll”. Tissues were fixed in 4% formalin, washed with PBS, embedded in paraffin, and cut into 5 μM-thick sections and stained with H&E.

For immunohistochemistry, tissue sections were deparaffinized, rehydrated and washed with PBS. Antigen retrieval was performed using Epitope Retrieval Solution, pH 6.0 (Leica Biosystems). Sections were incubated overnight at 4°C with CD8a (Cell signaling technology, D4W2Z, dilution: 1:500), CD11c (Cell signaling technology, D1V9Y, dil: 1:200), F4/80 (Cell signaling technology, D4C8V, dilution: 1:500), NK1(Cell signaling technology, E6Y9G, dilution: 1:150), in Dako antibody diluent. Slides were then washed three times with PBS and incubated for 2h at room temperature with Alexa Fluor 594-conjugated and Alexa Fluor 488-conjugated secondary antibodies. Nuclei were stained with SlowFade Gold Antifade reagent (Vector) with 4′,6-diamidino-2-phenylindole (DAPI, Vector). Data were obtained using Olympus TH4-100 and Leica DMi8 Fluorescence microscopes.

#### Tumor digestion

Tumors were harvested at day 12 after tumor injection and digested with 100 μg/mL DNase I (Sigma) and 1mL of collagenase D (Roche) at 37°C for 1 h. To generate a single-cell suspension, the digested material was passed through a 70μm cell strainer. The cells were then washed two times with phosphate-buffered saline (PBS) and incubated with the indicated antibodies for flow cytometry.

#### Flow cytometry

Tumor single cells were washed with FACS staining buffer, before incubated with the indicated antibodies (dilution 1:200) at 4°C for 20 min. Cells were then fixed using 1% of formaldehyde in PBS (for 20 min), followed by two washes before resuspended in FACS staining buffer. The following antibodies were used: NK1.1 (Clone: OK136), CD25 (Clone: 3C7), CD8 (Clone: 53–6.7), CD4 (Clone: RM4-5), Lag3 (Clone: C9B7W), CD69 (Clone: H1.2F3), B220 (Clone: RA3-6B2), CD44 (Clone: IM7), CD45 (Clone: 104), CD279 (Clone: 29F.1A12), MHC II (Clone: M5/114.15.2), CD11b (Clone: M1/70), PDCA-1 (Clone: 129c1), CD11c (Clone: N418), MHC I (Clone: AF6-88.5), F4/80 (Clone: BM8), CD206 (Clone: C068C2), GR1 (Clone: RB6-8C5), CD80 (Clone: 16-10A1). All the data were collected on an BD FACSymphony A5 and analyzed using Flowjo Software (10.8.2).

#### Spatial processing

Processing of samples: Formalin fixed paraffin embedded Tissue Micro Array (TMA) were used. Mouse samples were profiled using *in situ* Xenium platform (10x Genomics). Samples were mounted onto the Xenium slides, followed by fixation of the samples according to the manufacturer’s protocol (Xenium *in Situ* - FFPE Tissue Preparation CG000578 | Rev F). Target transcripts were hybridized with Xenium Mouse Tissue Atlassing predesigned Gene expression probes (Xenium *in Situ* Gene Expression CG000749 | Rev B). After probe hybridization, ligation and amplification were performed, and the tissues were stained with cell segmentation markers overnight, followed by nuclear staining (DAPI). The slides were then loaded onto the Xenium Analyzer. Following imaging, the slides were stained for H&E (Xenium Post-Xenium Analyzer H&E Staining CG000613 |B).

TMA core extraction and cell segmentation: Whole-slide DAPI images were loaded into QuPath (v0.6.0), where rectangular regions of interest (ROIs) were manually annotated around each TMA core. Core boundary coordinates (in microns) were exported using a custom Groovy script that converts pixel coordinates to physical units using the slide’s pixel calibration. Raw Xenium transcript tables were spatially subset per core using these coordinate boundaries. Transcripts with quality value (QV) < 20 were excluded, along with BLANK, NegControl, and UnassignedCodeword probes, yielding 183.7 million quality-filtered transcripts across the 14 cores. Cell segmentation was performed using Baysor (v0.7.1) on per-core transcript CSV files with Xenium-assigned cell IDs provided as prior segmentation labels.

Quality control and processing: Baysor outputs were loaded and concatenated into a single AnnData object. Quality filtering removed cells with fewer than 5 transcripts or fewer than 3 unique genes, retaining 504,862 cells across 379 genes from an initial 506,105 Baysor-segmented cells (1,243 cells removed). Raw transcript counts were stored in a separate layer, and expression values were normalized to counts per 10,000 followed by log1p transformation.

Batch integration and dimensionality reduction with scVI: Batch effects across slides were corrected using scVI (scvi-tools v1.3.0). A variational autoencoder was trained with the following parameters: 10 latent dimensions, 128 hidden units, 1 encoder/decoder layer, zero-inflated negative binomial (ZINB) gene likelihood, maximum KL divergence weight of 1.0, and FP16 (mixed-precision) with batch size of 2048. The 10-dimensional scVI latent representation was used for all downstream analyses.

Unsupervised clustering and cell type annotation: A k-nearest neighbor graph (k = 15) was constructed on the scVI latent space using rapids_singlecell (v0.12.1). UMAP visualization, Leiden clustering, and marker gene identification were performed using scanpy (v1.10.4). UMAP embeddings were generated with min_dist = 0.1, spread = 1.0, and spectral initialization. Community detection used the Leiden algorithm at resolution 0.5. Marker genes were identified via Wilcoxon rank-sum test, retaining genes with adjusted *p*-value <0.05 and >30% non-zero expression in the test group to guide cell type annotation.

Iterative subclustering and doublet removal: Each major cell type compartment was independently subclustered using the same scVI→neighbors→UMAP→Leiden pipeline (identical hyperparameters as above). Iterative rounds of sub clustering were performed per compartment with doublets, multiplets, and contaminating populations identified by aberrant co-expression of lineage-specific markers and removed at each round.

Spatial niche identification: Spatial cellular niches were identified using Cell Charter (v0.3.5). A spatial neighbor graph was constructed for each TMA core using 10 nearest neighbors based on cell centroid coordinates. Neighborhood representations were computed by aggregating scVI latent embeddings over 1-hop and 2-hop spatial neighbors. The optimal number of niches was determined using Cell Charter’s ClusterAutoK module, which evaluates cluster stability across 20 independent runs for each k in the range 5–20.

Calculation of TMA core areas: Images were imported into QuPath for analysis using standard Hematoxylin and Eosin (H&E) stain vectors. Individual TMA cores were identified and labeled using the automated TMA dearraying tool. Parameters for core detection included a core diameter of 2.0mm, a density threshold of 5.0, and a bounds scale factor of 105.0. Following automated detection, core boundaries were manually refined where necessary. To calculate the precise tissue area within each core, a machine learning-based pixel classifier was developed. Representative regions of tissue, background, and border were manually annotated across multiple cores to create a robust training set. An artificial neural network (ANN_MLP) classifier was utilized, trained, and applied at a pixel size of 4.0μm/px. The trained classifier was applied to all detected cores. The total area for each core expressed in μm2 was automatically calculated based on the calibrated pixel size within the image metadata. Data were exported using the “Measurement Maps” feature for downstream analysis.

Differential cell density analysis: To test for differences in cell type, sub type and spatial niche density between treatment groups, we used edgeR’s (v4.4.0) generalized linear model (GLM) quasi-likelihood (QL) framework. Cell type counts per core were organized into a count matrix (cell types x cores). Tissue area per core (mm^2^) was used as the library size (equivalent to a log-area offset), allowing direct modeling of cell densities. A design matrix encoding treatment group was specified with Ctrl as the reference level. Dispersions were estimated via glmQLFit with robust = TRUE. Differential density was assessed using the QL F-test. *p*-values were corrected for multiple testing using the Benjamini–Hochberg (BH) method.

Compositional analysis: Compositional differences in cell type, sub type and spatial niche proportions between treatment groups were assessed using sccomp (v2.1.17) using the sum-constrained Beta-Binomial model. The model was specified with formula ∼0 + group, and the contrast groupCtrl - groupIndole was tested. Outlier cells were identified and removed using sccomp_remove_outliers. Posterior probabilities of the null hypothesis (c_pH0) and BH-corrected false discovery rates were computed for each comparison.

#### Bacterial DNA extraction

Mouse fecal pellets were collected at day 0 (before tumor cell injection), and then at days 20 and 30 after tumor cell injection. Samples were frozen on dry ice and stored at −80°C. Bacterial DNA was extracted using the QIAmp Fast DNA Stool Mini Kit (Qiagen).

#### Analysis of Bacteroides

To identify bacterial strain that is similar to *B. rodentium,* we performed phenotypical and genomic comparison between different *Bacteroides strains.* We analyzed genomes from BV-BRC public database (https://www.bv-brc.org/). All *Bacteroides uniformis* analyzed were found to carry the TnaA enzyme (fig|411479.10.peg.1603 | BACUNI_01438 | Tryptophanase (EC 4.1.99.1), https://www.bv-brc.org/view/Feature/PATRIC.411479.10.NZ_DS362241.CDS.100381.101754.fwd and the ArAT enzyme (fig|411479.10.peg.506 | BACUNI_00099 | Aspartate/aromatic aminotransferase (EC 2.6.1.1), https://www.bv-brc.org/view/Feature/PATRIC.411479.10.NZ_DS362229.CDS.18972.20090.fwd#view_tab=overview.

#### 16S bacterial rRNA gene sequencing data processing

To determine bacterial composition, the V3/4 variable region of the 16 S rRNA gene was amplified using 16S-341F (CCTACGGGNGGCWGCAG) and 16S-785 R (GACTACHVGGGTATCTAATCC) primers.

Sequencing of pooled libraries was carried out using Illumina Nextera platform. Sequencing data were processed using Qiime2 pipeline. Raw sequencing data were processed to remove primers using cutadapt followed by denoising, merging, chimera removal to generate unique amplicon sequence variants (ASV) using the Dada2 plugin. For taxonomic assignments of these ASVs, first naive Bayes classifiers was generated on extracted V34 sequences obtained from SILVA 138.1 database using fit-classifier-naive-bayes plugin. Finally, this trained classifier was used to assign the taxonomy to the representative sequences of each ASV using feature-classifier classifiers learn plugin. Also, these representative sequences were aligned using MAFFT and phylogenetic trees both rooted and unrooted were constructed using FasTree.

Generated ASVs along with the taxonomies were used for the downstream analysis. Firstly, contaminants were identified and removed based on the prevalence of taxa in the negative control samples. Then additional filtering was done by removing ASV if it is not present in at least 10% of the samples (separately for both group; for MC the cut-off is 8 samples and for TRP the cut-off is 5 samples) followed by removing samples if they do not have sequencing depth of at least 1000 sequences. This final filtered ASV table was used for downstream statistical analysis.

Microbial community analyses were performed with the R statistical interface. Briefly, for alpha diversity, beta diversity, permutational multivariate analysis of variance (PERMANOVA), multiple R packages such as vegan, ape, phyloseq were used. For alpha diversity analysis, microbial richness was considered by identifying observed ASVs across samples and for beta diversity bray-curtis distances among samples were computed using relative abundances of each ASV in the sample. Taxonomic distribution was shown using plot bar function using phyloseq package in R. Finally, variance partition analysis was used to identify the variables which are contributing the maximum variance in the data and to detect signature taxa with maximum variance contribution. Distribution of top taxa based on the treatment were plotted separately. Kruskal–Wallis tests were used to check the statistical significance among multiple groups using the kruskalmc function of pgirmess package in R. All graphs were plotted using ggplot function in R.

#### BMDC stimulation with *B. rodentium and B. uniformis* supernatants

Bone marrow was isolated from tibiae and femurs of C57BL/6J mice and cultured 8 days in RPMI 1640 media containing 10% FBS, 1% penicillin/streptomycin, and recombinant mouse GM-CSF (20 ng/mL, BioLegend) at 37°C.

#### Detection of serum cytokines and chemokines

Serum was collected before mice were euthanized and stored at −80^0^C. Cytokines and chemokines were quantified using the Bio-Plex Pro Mouse Cytokine 23-plex Assay kit (BIO-RAD). All data were collected using Luminex XMAP Technology, and a MAGPIX instrument and analyzed with luminex xPONENT software.

### Quantification and statistical analysis

Statistical analyses were performed using Prism software (version 10, GraphPad). Differences between two groups were compared using a two-tailed unpaired *t* test for parametric data or the Mann-Whitney U test for non-parametric data. Tumor growth curves were analyzed using two-way ANOVA. Statistical details of experiments can be found within the figure and/or figure legends. Unless otherwise indicated in the figure legend or text, correction of multiple comparisons was performed utilizing the Benjamini-Hochberg procedure as previously described. All tests were two-sided.

## Supplemental information

Document S1. Figures S1–S6 and Tables S1, S2, and S5

Table S3. List of metabolites in cecal samples from mice colonized with ASF or ASF plus *B. rodentium*, related to Figure 3 The table includes in the column the index (a unique identifier for each detected compound in the dataset), compounds (name of the metabolite assigned to that GC-MS peak), Class I (a broad chemical category), Class II (a more specific subclass of Class I); Q1 (Da) (the mass-to-charge ratio [m/z] of the ion selected in the first quadrupole [Q1]); molecular weight (Da) (the theoretical molecular mass of the compound); ionization model (how the molecule was ionized in the mass spectrometer); formula (chemical formula of the compound), and level (the confidence level of the compound identification) of each metabolite analyzed. Green, the measure of the metabolites associated with the ASF group; orange, the measure of the metabolites associated with ASF plus *B. rodentium* group (6 mice per group).

Table S4. Binary phenotype matrix comparison of *B. rodentium* and other *Bacteroides* strains, related to Figure 4 This table compares the phenotypic metabolites of each *Bacteroides* strains, compared with *B. rodentium.* In the column, we list the different metabolites that were analyzed. 1 (green) marks the metabolite is present or positive result, 0 (orange) marks the metabolite is absent or negative result, and intermediate (e.g., 0.7) indicates a partial or weak expression.

Document S2. Article plus supplemental information
