# Supplementary material for: Tryptophan degradation by intestinal Bacteroides induces anti-tumor immunity and limits melanoma growth
Source: Cell Rep Med. 2026 Jul 14;7(7):102921. doi: 10.1016/j.xcrm.2026.102921 (PMC13400165; doi:10.1016/j.xcrm.2026.102921)
Supplement: Document S1. Figures S1–S6 and Tables S1, S2, and S5 [file mmc1.pdf]

Cell Reports Medicine, Volume *7*

Supplemental information

Tryptophan degradation by intestinal *Bacteroides* induces anti-tumor immunity and limits melanoma growth

Ximena Diaz Olea, Kristin Beede, Gabriel Pereira, David Scott, Christopher Petucci, Eric Martens, Dmitri Rodionov, Aagam Shah, Miguel P. Martinez, Hyungsoo Kim, Ashok Kumar Sharma, Anthony Martin, Tongwu Zhang, Mark B. Faries, Omid Hamid, Suzanne Devkota, Andrei Osterman, Simon Knott, Emile E. Voest, Nadim J. Ajami, Jennifer Wargo, Amanda E. Ramer-Tait, and Ze'ev A. Ronai

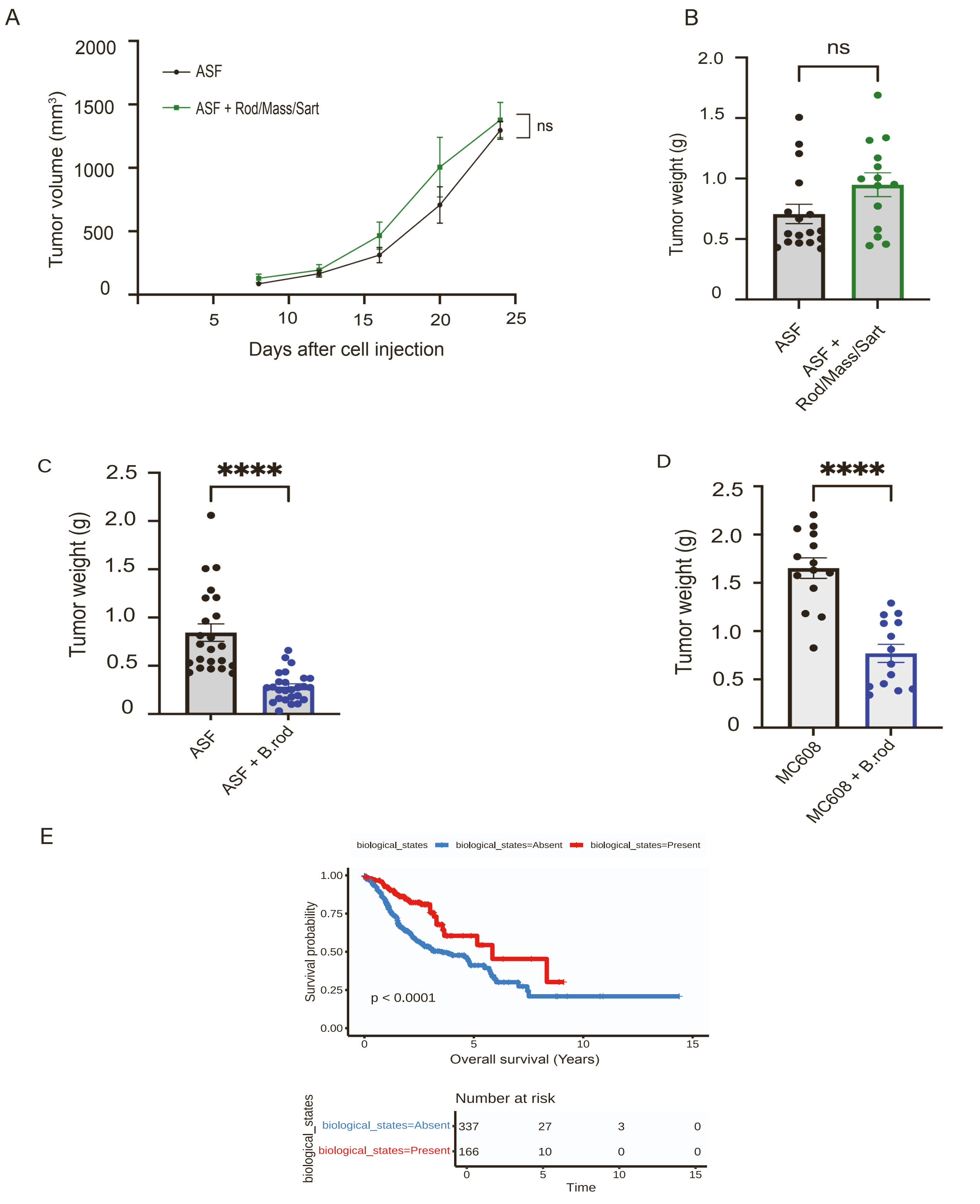

Supplementary Figure 1: *B. rodentium* colonization limited tumor growth. Growth (A) and weight (B) of tumors in germ-free (GF) mice colonized with either ASF or ASF plus *B. rodentium*, *P. sartorii*, and *P. massiliensis* 14 days prior to YUMM1.5 tumor cell injection. (C) Weight of YUMM1.5 tumors established in GF mice colonized with either ASF or ASF plus *B. rodentium* by oral gavage 14 days prior to YUMM1.5 tumor cell injection (n = 15 mice/treatment; data represent two experiments). (D) Weight of YUMM1.5 tumors established in GF mice colonized with either microbiome MC608-F-a1 or MC608-F-a1 plus *B. rodentium* by oral gavage 14 days prior to YUMM1.5 tumor cell injection (n=18 mice/treatment; data represent two experiments). (E) Kaplan-Meier survival analysis (years) reflecting the relative abundance of the microbiome genus *Bacteroides* for overall patient survival. Biological state indicates the presence (positive) or absence (negative) of *Bacteroides* in the analysis. The log-rank test p-value is indicated in the survival plot. Cox proportional hazards model results: HR = 0.50 (95% CI: 0.33– 0.75), p = 0.001. Data were analyzed by unpaired t-test represented as +/- SEM, NS - not significant;. *P < 0.05, **P < 0.005, ***P < 0.001, ****P < 0.0001 using two-tailed t-test or two-way ANOVA. Relate to Figure 1

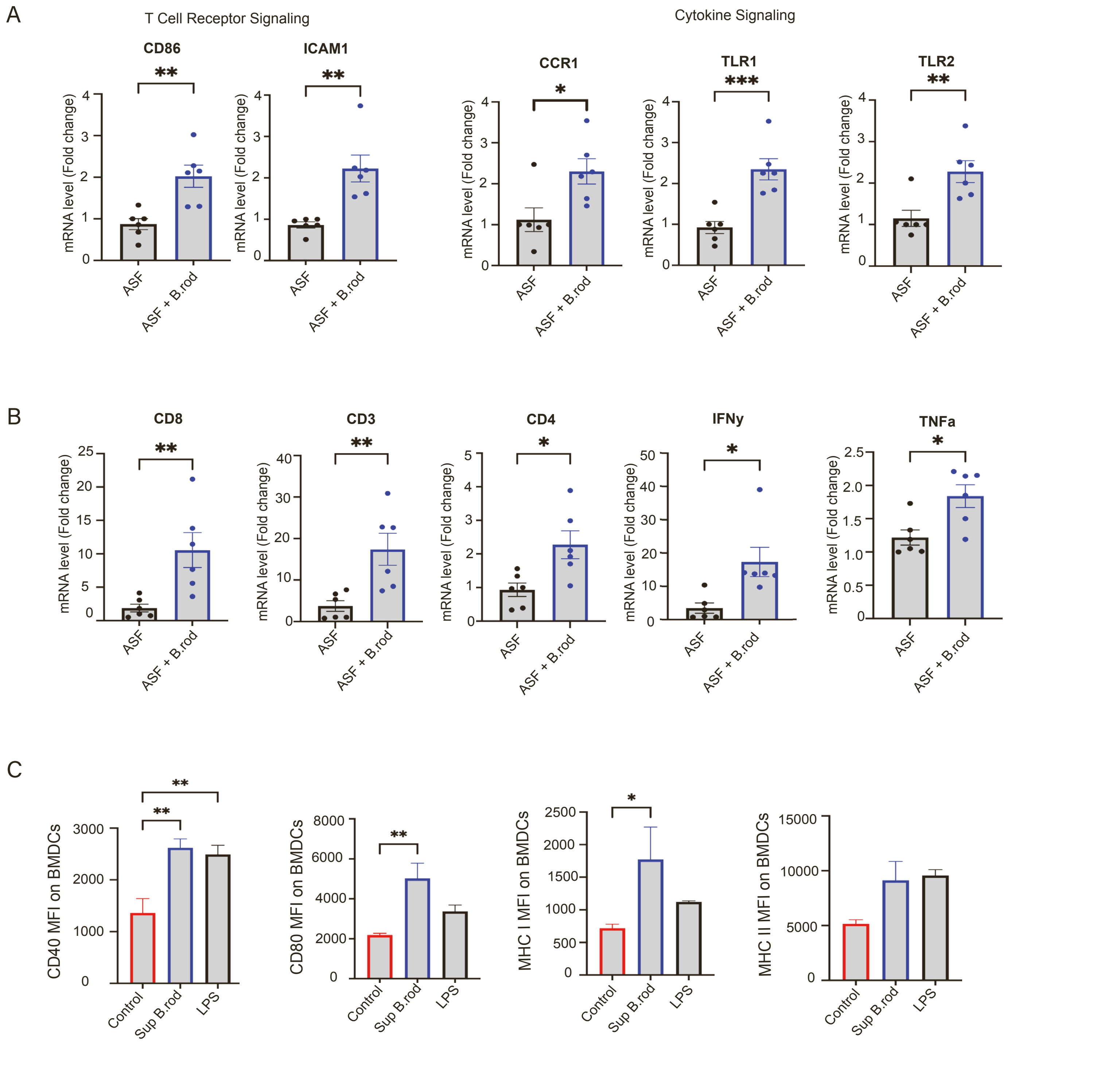

Supplementary Figure 2: Upregulated immune signaling in mice colonized with *B. rodentium*. (A) RT-qPCR validation of key genes functioning in anti-tumor immunity as initially identified by RNAseq. Data shown represent three analyses from each of two experiments. (B) RT-qPCR validation of RNAseq data of genes implicated in immune signaling (ASF, n=3; ASF plus *B. rodentium*, n=3; two independent experiments). (C) CD40, CD86, MHC I, and MHC II expression (MFI) on bone marrow-derived dendritic cells (BMDCs) untreated (control), stimulated with 10% of secretome from *B. rodentium*, or treated with LPS (positive control) in vitro for 24 hours (n=3). Data were analyzed by unpaired t-test represented as mean +/- SEM, NS - not significant, *P < 0.05, **P < 0.005, ***P < 0.001, by two-tailed t-test. Relate to Figure 2

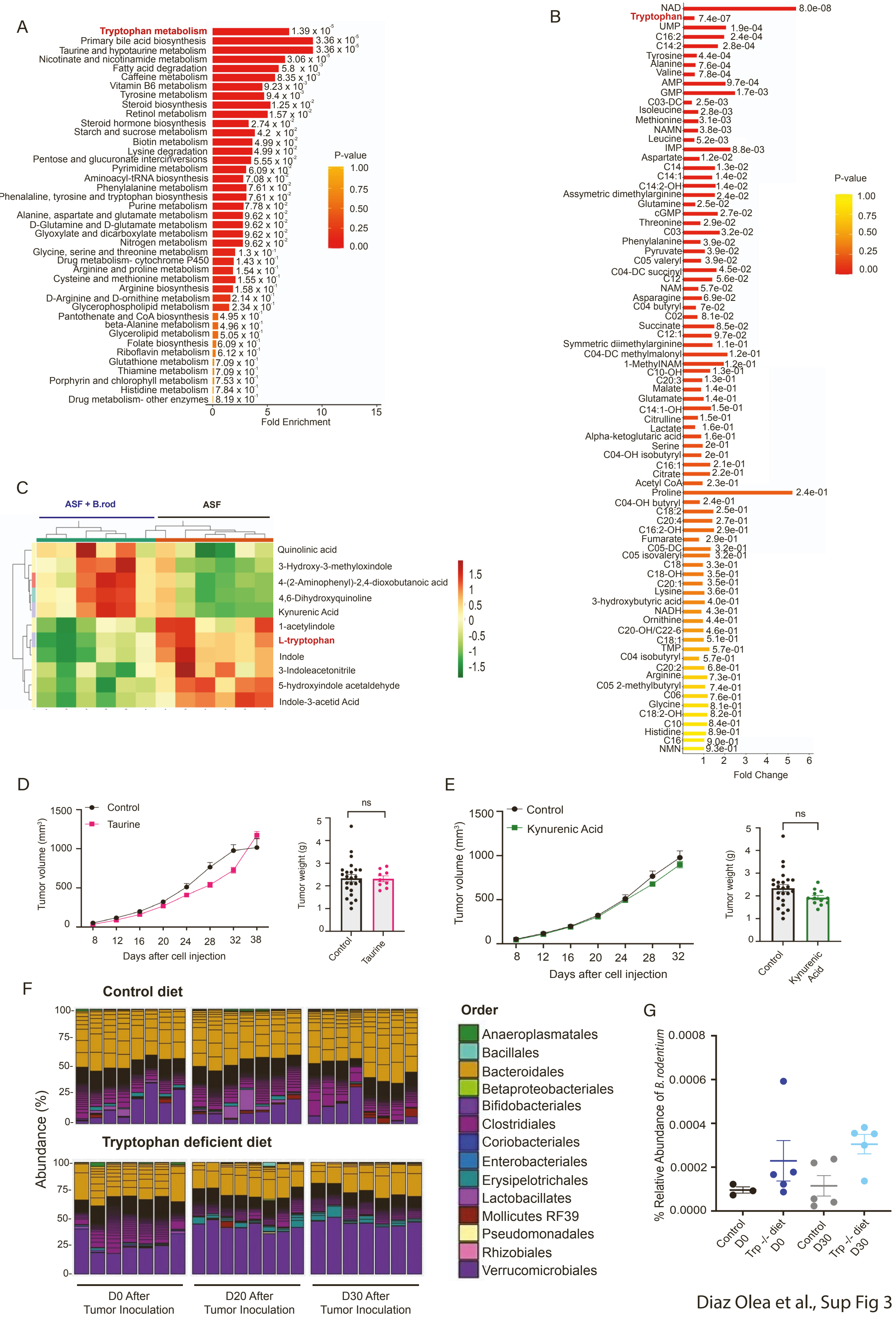

Diaz Olea et al., Sup Fig 3

Supplementary Figure 3: Metabolomic and sequencing analyses. (A) Independent metabolomic analysis depicts the top 50 metabolic sets ranked by P-value in KEGG pathway analysis (n=6 mice/treatment). (B) Metabolomic analysis showing metabolites ranked by P-value (ASF, n=3; ASF plus *B. rodentium,* n=3; two independent experiments). (C) Independent metabolic analysis reflected in heatmap depicting clustering of metabolites enriched in indicated groups, based on KEGG pathway analysis (n=6 mice/treatment). (D) Effects of taurine on melanoma tumor growth based on analysis of tumor size. Taurine was provided two weeks prior to subcutaneous injection of conventional C57BL/6J mice with YUMM1.5 cells (Control, n=22; Taurine n=10). (E) Effects of kynurenic acid provided three days prior to injection of conventional C57BL/6J mice with tumor cells on melanoma tumor growth (Control, n=22; kynurenic acid n=12). (F) Abundance of the taxonomic distribution across indicated samples based on analysis of 16S rRNA bacterial gene sequencing analysis of fecal samples from the indicated groups. This analysis provided the basis for more detailed analyses of select bacterial strains that were altered in this experiment (Figure 3C). (G) Percentage of relative abundance of *B. rodentium* in stool samples from WT mice administrated with normal diet or deficient tryptophan diet, at day 0 and 30 after tumor inoculation (n=5 mice per treatment). Data were analyzed by unpaired t-test represented as mean +/- SEM, NS- not significant; *P < 0.05 **P < 0.005, ***P < 0.001, ****P < 0.0001 by two-tailed t-test or two-way ANOVA. Relates to Figure 3.

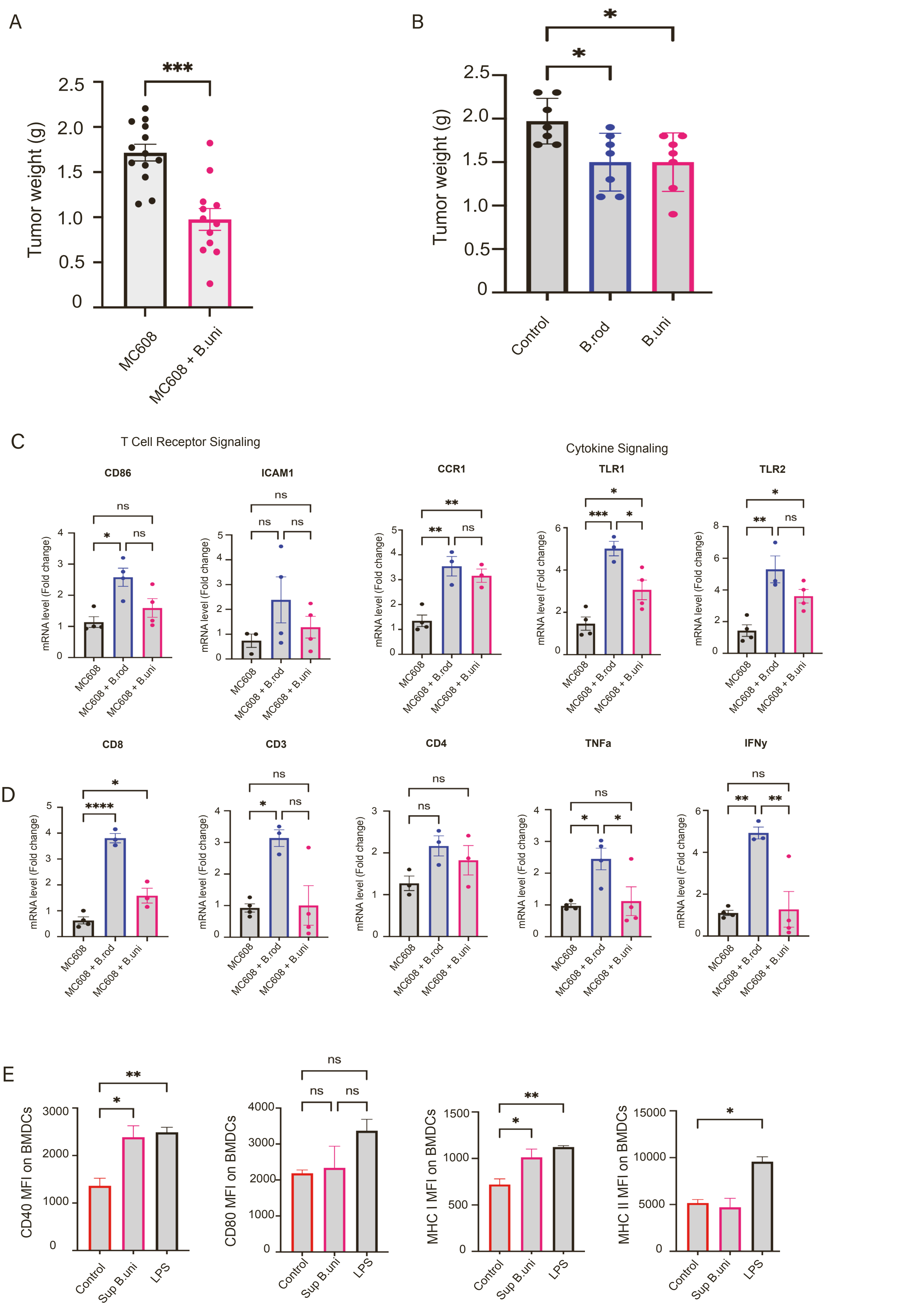

Diaz Olea et al., Sup Fig 4

Supplementary Figure 4: *B. uniformis* colonization limited tumor growth. (A) Weight of YUMM1.5 tumors in GF mice colonized with either microbiome MC608-F-a1 or MC608-F-a1 plus *B. uniformis*. (B) Weight of YUMM 1.5 tumors in conventional C57BL/6J mice colonized with either *B. rodentium* or *B. uniformis.* (C) RT-qPCR analysis validating key genes identified by RNAseq in GF mice colonized with MC608-F-a1, MC608-F-a1 plus B. rodentium or MC608-F-a1 plus *B. uniformis* (n=4 mice/treat-ment). (D) RT-qPCR analysis of genes identified by RNAseq as implicated in control of immune signaling in GF mice colonized with MC608-F-a1, MC608-F-a1 plus *B. rodentium*, or MC608-F-a1 plus *B. uniformis* (n=4/treatment). (E) CD40, CD86, MHC I, and MHC II expression (MFI) on bone marrow-derived dendritic cells (BMDCs) untreated (control), stimulated with 10% of secretome from *B. uniformis*, or treated with LPS (positive control) in vitro for 24 hours (n=3). Data shown represent two experiments. Data were analyzed by unpaired t-test represented as mean +/- SEM, NS - not significant; *P < 0.05 **P < 0.005, ***P < 0.001, ****P < 0.0001 by two-tailed t-test. Relates to Figure 4.

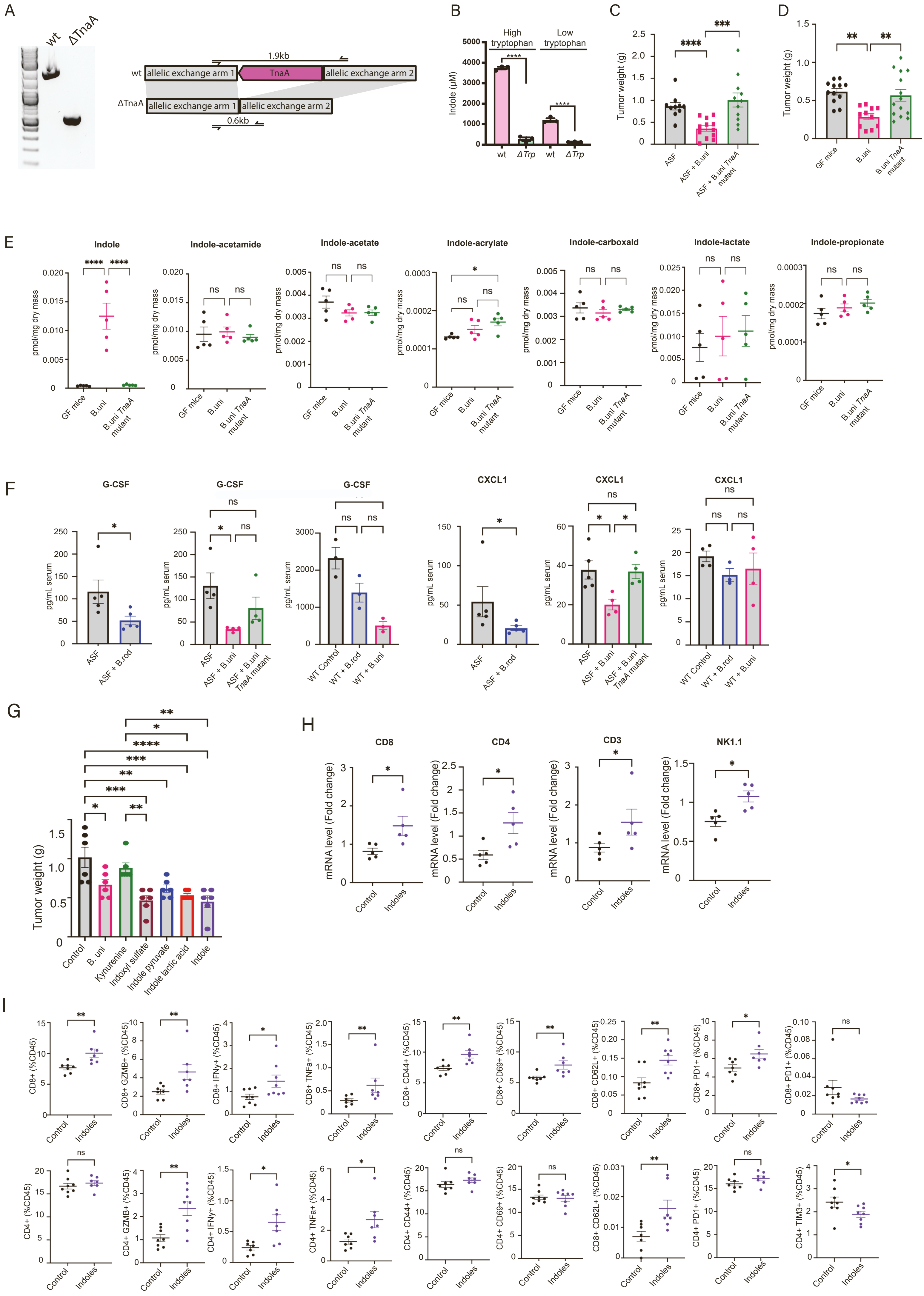

Diaz Olea et al., Sup Fig 5

Supplementary Figure 5: B. uniformis colonization limited tumor growth depending on the tryptophanase (TnaA) gene. (A) PCR validation of mutant tryptophanase in B. uniformis, and graphic abstract of the deletion of tryptophanase gene by allelic exchange (B) Validation of mutant tryptophanase activity in B. uniformis was assessed in vitro in high- or low-tryptophan media. (C) Weight of YUMM 1.5 tumors in GF mice that were colonized with either ASF, ASF plus B. uniformis, or ASF plus B. uniformis tryptophanase (TnaA) mutant. (D) Weight of YUMM 1.5 tumors in GF mice that were colonized with either B. uniformis or B. uniformis TnaA mutant. (E) Quantification of indicated indoles metabolites in cecal samples from GF mice colonized with B. uniformis or B. uniformis tryptophanase mutant (n = 5 mice/treatment). (F) Serum cytokines levels in GF mice colonized with ASF, ASF plus B. rodentium (n=5 mice/treatment) or ASF, ASF plus B. uniformis, ASF plus B. uniformis TnaA mutant (n=5 mice/treat-ment); conventional C57BL/6J mice, colonized with either B. rodentium or B. uniformis (n=4 mice/treatment). (G) Weight of YUMM 1.5 tumors in conventional C57BL/6J mice that were treated with B. uniformis, kynurenine, indoxyl sulfate, indole pyruvate, indole lactic acid or indole. (H) RT-qPCR of genes implicated in immune signaling (Control, n=3; Indoles, n=3; two independent experiments). (I) Quantification of tumor infiltration of CD8+ and CD4+ T cells, CD44+, CD69+, CD62L+, TIM 3+, PD1+, GZMB+, TNFa+ and IFNg+ on CD4+ and CD8+ T cell 12 days after injection of YUMM1.5 cells into conventional C57BL/6J mice treated with indoles (daily via oral gavage starting one day after tumor inoculation until end of the experiment), (n =8 mice/treat-ment). Data were analyzed by unpaired t-test represented as mean +/- SEM. NS- not significant; *$P < 0.05$, **$P < 0.005$, ***$P < 0.001$, ****$P < 0.0001$ by two-tailed t-test or two-way ANOVA. Relates to Figure 5.

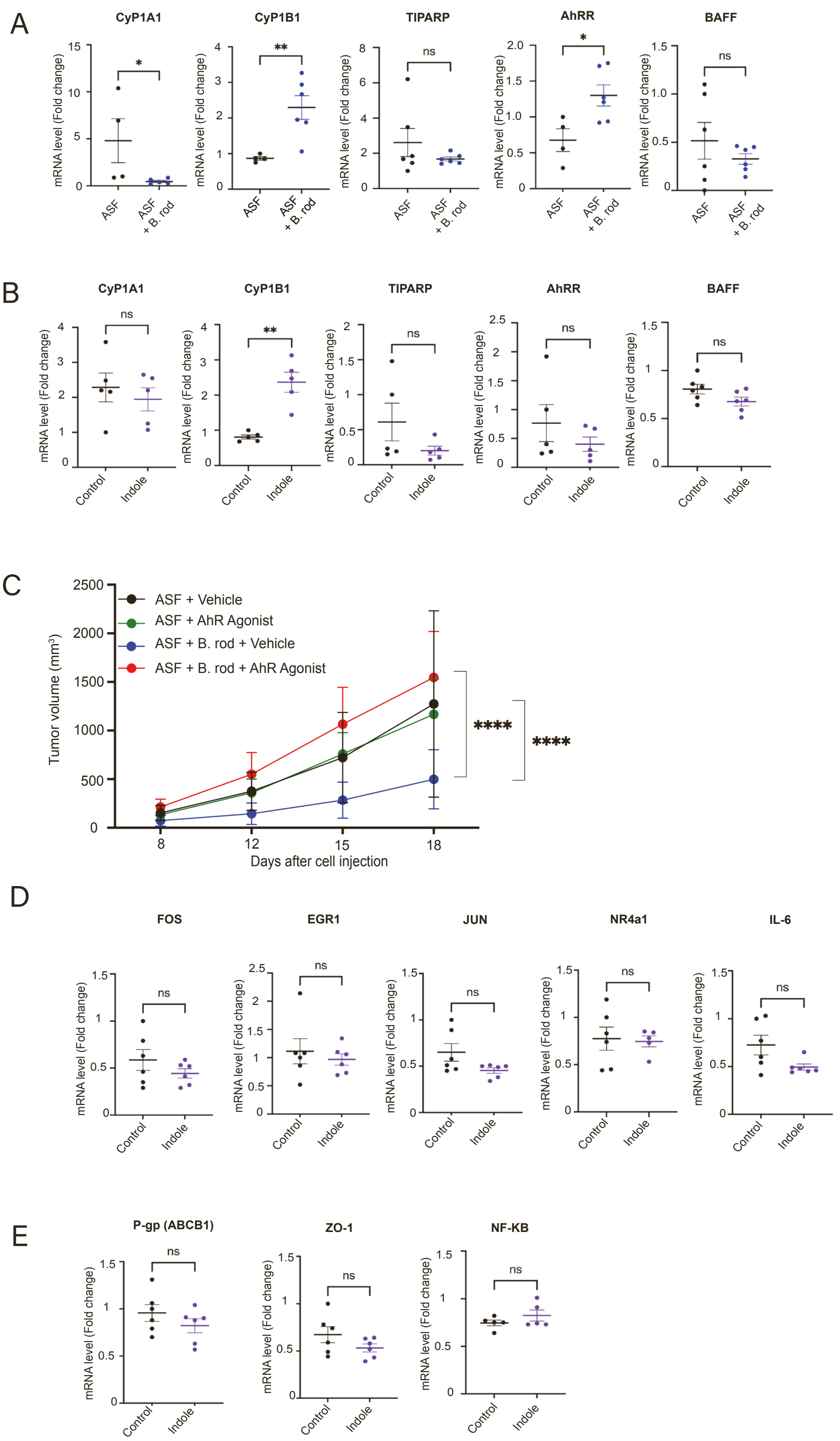

Supplementary Figure 6: Inhibition of tumor growth by B. rodentium or indole administration is not depending on the AhR, PXR and GPCR pathways. (A) RT-qPCR of genes implicated in AhR signaling (ASF, n=6; ASF plus B. rodentium, n=6). (B) RT-qPCR of genes implicated in AhR signaling (Control, n=5; Indoles, n=5). (C) Tumor growth in GF mice colonized with either ASF, ASF plus B. rodentium, 14 days prior to YUMM1.5 tumor cell injection, treated with AhR agonist (TCDD) or vehicle.(n = 15 mice/treatment). (D) RT-qPCR of genes implicated in GPCR signaling (Control, n=5; Indoles, n=5) (E) RT-qPCR of genes implicated in PXR signaling (Control, n=5; Indoles, n=5). Data were analyzed by unpaired t-test represented as mean +/- SEM; NS - not significant; *P < 0.05, **P < 0.005, ***P < 0.001, ****P < 0.0001 by two-tailed t-test or two-way ANOVA. Relates to Figure 6.

Supplemental Table 1: List of Primers used for qRT-PCR analysis.
Related to Figure 2

**Gene**	**Forward (5'-3')**	**Reverse (5'-3')**
CD86	TGTTTCCGTGGAGACGCAAG	TTGAGCCTTTGTAAATGGGCA
ICAM	GTGATGCTCAGGTATCCATCCA	CACAGTTCTCAAAGCACAGCG
CCR1	CTCATGCAGCATAGGAGGCTT	ACATGGCATCACCAAAAATCCA
TLR1	TGAGGGTCCTGATAATGTCCTAC	AGAGGTCCAAATGCTTGAGGC
TLR2	GCAAACGCTGTTCTGCTCAG	AGGCGTCTCCCTCTATTGTATT
TNFα	CCCTCACACTCAGATCATCTTCT	GCTACGACGTGGGCTACAG
IFNγ	ATGAACGCTACACACTGCATC	CCATCCTTTTGCCAGTTCCTC
CD3	ACTGTAGCCGAGAGAAATAAAGC	TGCCAGATTCGATGTGTTTT
CD4	AGGTGATGGGACCTACCTCTC	GGGGCCACCACTTGAACTAC
CD8	CCGTTGACCCGCTTTCTGT	CGGCGTCCATTTTCTTTGGAA
H3A	ATTGTGCCACATCGTGTATGG	GTGATGCAACATGCTTCGCA

Supplemental Table 2: Standards used for Gas Chromatography-Mass Spectrometry (GC-MS) Analysis.

Related to Figure 3

Name	RT	Mass	Product Mass	Collision Energ	Polarity
Pyruvate	3.69	160.1	75.1	10	Positive
Pyruvate	3.69	216.2	160.1	8	Positive
Octanoate	4.352	131.1	75	8	Positive
Octanoate	4.352	201.1	131.1	8	Positive
Lactate	4.5	261.2	189.2	6	Positive
Lactate	4.5	261.2	233.2	6	Positive
KIVA	4.92	188.1	75.1	10	Positive
KIVA	4.92	244.2	131.1	6	Positive
Picolinate	5.37	180.1	78	22	Positive
Picolinate	5.37	180.1	136.1	12	Positive
Picolinate	5.37	222.1	180.1	8	Positive
Alanine	5.38	260.2	158.2	14	Positive
Alanine	5.38	260.2	232.2	8	Positive
Nicotinate	5.4	180.1	106.1	14	Positive
Nicotinate	5.4	180.1	136.1	8	Positive
Glycine	5.85	246.2	103.1	10	Positive
Glycine	5.85	246.2	218.2	6	Positive
Sarcosine	6.49	260.2	158.2	10	Positive
Sarcosine	6.49	260.2	232.2	8	Positive
3-Hydroxybutyrate	6.62	275.2	159.2	6	Positive
3-Hydroxybutyrate	6.62	275.2	233.2	6	Positive
2Aminobutyrate	6.72	274.2	142.1	8	Positive
2Aminobutyrate	6.72	274.2	246.2	8	Positive
BetaAlanine	7.36	260.2	117	10	Positive
BetaAlanine	7.36	260.2	218.2	8	Positive
3-phenylpropionate	7.535	91.1	65	14	Positive
3-phenylpropionate	7.535	207.1	91	16	Positive
Urea	7.62	231.2	173.1	8	Positive
Valine	7.65	288.2	156.1	6	Positive
Valine	7.65	288.2	260.2	8	Positive
Norvaline	7.93	288.2	186.2	14	Positive
Norvaline	7.93	288.2	260.2	8	Positive
Decanoate	8.189	131.1	75	8	Positive
Decanoate	8.189	229.2	95.1	8	Positive
Decanoate	8.189	229.2	131.1	8	Positive
Leucine	8.46	302.2	274.2	8	Positive
Nicotinamide	8.6	179.1	105.1	20	Positive
Nicotinamide	8.6	179.1	136.1	10	Positive
Ethylmalonate	8.91	303.2	189.2	6	Positive
Isoleucine	9.03	200.2	144.2	8	Positive
Isoleucine	9.03	274.2	131.1	28	Positive
GABA	9.56	258.2	216.1	10	Positive
GABA	9.56	274.2	258.2	6	Positive
Putrescine	9.56	259.2	128.2	10	Positive
Putrescine	9.56	259.2	184.2	14	Positive
Putrescine	9.56	316.3	259.2	6	Positive
Proline	9.58	258.2	131.1	28	Positive
Proline	9.58	286.2	258.2	8	Positive
Succinate	9.62	289.2	189.2	6	Positive
Uracil	9.69	283.2	99.1	18	Positive
Uracil	9.69	283.2	241.2	8	Positive
Itaconate	10.18	301.1	189.1	6	Positive
Fumarate	10.2	287.2	245.1	8	Positive
Cadaverine	11.281	142.1	59	18	Positive
Cadaverine	11.281	184.2	128.1	10	Positive
Cadaverine	11.281	273.2	84.1	8	Positive
Glutarate	11.366	303.1	245.2	5	Positive
Glutarate	11.366	303.1	275.2	5	Positive
Taurine	12.18	296.2	182.1	6	Positive
Glycerate	12.99	231.2	115.1	6	Positive
Glycerate	12.99	391.2	231.2	6	Positive
Methionine	13.15	292.2	244.2	8	Positive
Methionine	13.15	320.2	292.2	8	Positive
Serine	13.52	362.3	230.2	8	Positive
Serine	13.52	390.3	362.3	8	Positive
Threonine	14	303.3	202.2	14	Positive
Threonine	14	404.3	376.3	8	Positive
Tryptophol	14.879	200.1	184.1	10	Positive
Tryptophol	14.879	218.1	144.1	14	Positive
Tryptophol	14.879	275.2	218.2	6	Positive
Phenylalanine	15.02	336.2	204.1	8	Positive
Phenylalanine	15.02	336.2	308.2	8	Positive
Hippurate	15.127	105.1	77	12	Positive
Hippurate	15.127	192.1	174.1	16	Positive
Hippurate	15.127	236.1	105	10	Positive
N-acetyl-aspartate	15.37	287.1	245.1	6	Positive
N-acetyl-aspartate	15.37	346.2	214.1	6	Positive
N-acetyl-aspartate	15.37	346.2	259.2	8	Positive
Tyramine	15.459	125.7	118.1	8	Positive
Tyramine	15.459	144.1	88.1	6	Positive
Tyramine	15.459	308.2	219.2	10	Positive
4OHBenzoate	15.47	265.2	151.1	20	Positive
4OHBenzoate	15.47	265.2	193.1	20	Positive
4OHBenzoate	15.47	309.1	235.1	18	Positive
4OHBenzoate	15.47	309.1	265.2	10	Positive
Malate	15.49	419.2	217.2	8	Positive
Malate	15.49	419.2	287.2	8	Positive
Malate	15.49	419.2	403.2	8	Positive
Citramalate	15.56	301.1	115.1	8	Positive
Citramalate	15.56	433.2	273.2	8	Positive
Indole-acetate	16.013	188.1	160.1	10	Positive
Indole-acetate	16.013	232.1	188.1	8	Positive
Indole-acetate	16.013	289.1	232.1	6	Positive
Aspartate	16.11	302.2	218.2	8	Positive
Aspartate	16.11	390.3	216.2	10	Positive
Aspartate	16.11	418.3	390.2	8	Positive
Tryptamine	16.15	144.1	88.1	6	Positive
Tryptamine	16.15	188.1	160.1	10	Positive
Tryptamine	16.15	217.1	188.1	14	Positive
Ketoglutarate	16.43	388.2	156.1	8	Positive
Ketoglutarate	16.43	388.2	228.2	8	Positive
Hydroxyproline	16.53	314.3	182.2	10	Positive
Hydroxyproline	16.53	416.3	388.3	8	Positive
Cysteine	16.8	302.2	218.2	6	Positive

Cysteine	16.8	378.2	246.2	8	Positive
Cysteine	16.8	406.2	378.2	8	Positive
2HG	17.02	273.2	245.2	6	Positive
2HG	17.02	433.3	245.2	10	Positive
2HG	17.02	433.3	273.2	8	Positive
Phosphoethanolamine	17.07	426.2	211.1	30	Positive
Phosphoethanolamine	17.07	426.2	269.2	20	Positive
Hypoxanthine	17.12	193.1	166.1	10	Positive
Hypoxanthine	17.12	307.2	193.1	16	Positive
PEP	17.24	259.2	189.2	6	Positive
PEP	17.24	453.2	259.2	10	Positive
NacetylGlutamate	17.4	228.1	186.1	8	Positive
NacetylGlutamate	17.4	360.2	228.1	8	Positive
NacetylGlutamate	17.4	360.2	318.2	8	Positive
Meglutol	17.419	273.2	199.2	12	Positive
Meglutol	17.419	447.3	241.2	6	Positive
Meglutol	17.419	447.3	273.2	6	Positive
Glutamate	17.82	330.3	170.2	10	Positive
Glutamate	17.82	432.3	272.2	10	Positive
Indole-propionate	17.827	130	77	24	Positive
Indole-propionate	17.827	246.1	117	6	Positive
Indole-propionate	17.827	303.1	130.1	20	Positive
Ornithine	17.88	286.2	154.1	10	Positive
Ornithine	17.88	286.2	258.2	8	Positive
Asparagine	18.22	400.2	326.2	8	Positive
Asparagine	18.22	417.3	285.2	6	Positive
Asparagine	18.22	417.3	400.3	8	Positive
Indole-carboxald	18.293	274.2	217.1	8	Positive
Indole-carboxald	18.293	330.2	217.1	14	Positive
Indole-carboxald	18.293	330.2	274.2	8	Positive
Indole-acetamide	18.427	130.1	77	24	Positive
Indole-acetamide	18.427	231.1	130	16	Positive
Indole-acetamide	18.427	288.2	231.1	6	Positive
Indole-carboxald2	19.168	274.2	217.1	8	Positive
Indole-carboxald2	19.168	330.2	217.1	14	Positive
Indole-carboxald2	19.168	330.2	274.2	8	Positive
Lysine	19.284	300.2	168.1	10	Positive
Lysine	19.284	300.2	272.2	8	Positive
Lysine	19.284	329.3	198.2	8	Positive
cAconitate	19.41	459.2	253.1	8	Positive
cAconitate	19.41	459.2	327.2	8	Positive
Glutamine	19.85	431.3	271.2	10	Positive
Glutamine	19.85	431.3	357.2	16	Positive
Glutamine	19.85	431.3	385.2	10	Positive
DHAP	21.12	452.2	211	30	Positive
DHAP	21.12	526.3	357.1	20	Positive
Vanillylmandelate	21.14	381.2	251.2	10	Positive
Vanillylmandelate	21.14	483.3	383.1	12	Positive
Vanillylmandelate	21.14	483.3	455.2	10	Positive
Dopamine	21.22	266.1	249.1	8	Positive
Dopamine	21.22	322.2	223.2	14	Positive
Dopamine	21.22	438.3	308.2	12	Positive
Dopamine	21.22	438.3	322.2	10	Positive
Glycerol3P	21.34	211	115	30	Positive
Glycerol3P	21.34	211	133	16	Positive
Glycerol3P	21.34	571.3	171.2	12	Positive
DHAP2	21.39	309.2	267.2	8	Positive
DHAP2	21.39	526.3	357.1	18	Positive
Spermidine	21.413	186.2	130.1	10	Positive
Spermidine	21.413	430.3	186.2	10	Positive
Spermidine	21.413	430.3	202.2	15	Positive
Kynurenate	21.472	246.1	202.1	12	Positive
Kynurenate	21.472	360.1	246.1	16	Positive
Kynurenate	21.472	402.2	242.2	14	Positive
Glycerol3P2	21.9	269.1	133	28	Positive
Glycerol3P2	21.9	269.1	211	10	Positive
Glycerol3P2	21.9	571.3	171.2	12	Positive
Kynurenine	21.93	379.2	219.1	12	Positive
Kynurenine	21.93	379.2	362.2	8	Positive
Histidine	21.94	196.2	140.1	8	Positive
Histidine	21.94	440.3	280.2	16	Positive
Histidine	21.94	440.3	412.3	10	Positive
Xanthine	22.29	265.1	131.1	26	Positive
Xanthine	22.29	265.1	158.1	20	Positive
Xanthine	22.29	437.2	363.2	12	Positive
Indole-lactate	22.304	130.1	77	24	Positive
Indole-lactate	22.304	348.2	216.1	12	Positive
Indole-lactate	22.304	376.2	348.3	8	Positive
Citrate	22.4	459.2	253.1	8	Positive
Citrate	22.4	591.4	459.2	8	Positive
3PG	22.48	269.1	211	10	Positive
3PG	22.48	269.1	253.1	5	Positive
3PG	22.48	585.3	259.2	10	Positive
Tyrosine	22.57	302.2	218.2	6	Positive
Tyrosine	22.57	466.3	438.3	10	Positive
Isocitrate	22.57	459.2	387.3	8	Positive
Isocitrate	22.57	591.3	403.2	12	Positive
Isocitrate	22.57	591.3	459.2	8	Positive
Tryptophan	23.09	302.2	218.2	5	Positive
Tryptophan	23.09	375.2	132.1	20	Positive
Tryptophan	23.09	375.2	347.2	10	Positive
Pantothenate	23.134	375.3	243.2	6	Positive
Pantothenate	23.134	504.3	199.2	10	Positive
Pantothenate	23.134	504.3	372.2	10	Positive
Serotonin	23.22	261.2	204.1	10	Positive
Serotonin	23.22	347.2	318.2	16	Positive
Serotonin	23.22	347.2	330.1	14	Positive
Indole-acrylate	24.618	284.1	170.1	12	Positive
Indole-acrylate	24.618	358.1	284.2	12	Positive
Indole-acrylate	24.618	415.2	358.2	8	Positive
Spermine	25.689	186.2	130.1	5	Positive
Spermine	25.689	374.3	243.3	5	Positive
Spermine	25.689	487.4	243.3	5	Positive
5-hydroxytryptophan	25.755	260.1	188.1	22	Positive
5-hydroxytryptophan	25.755	505.3	477.3	10	Positive

Supplemental table 5: List of genomes analyzed in BV-BRC public database.

Related to Figure 4.

LEGEND TO SUPPLEMENTAL TABLE 5
The BV-BRC public database (https://www.bv-brc.org/) was used to analyze genome of indicated bacterial strains. The table include ID (the unique genome identifier in BV-BRC public database); Genus (the genus name of the organism); Species (the species name, more specific than genus); MC (name of the bacterial strain); Similarity (normalized value representing how similar the genome is to *B. rodentium)*.

ID	Genus	Species	mc	Similarity
1236512.8	Bacteroides	Bacteroides rodentium	Bacteroides rodentium CM 16496	1
691816.5	Bacteroides	Bacteroides rodentium	Bacteroides rodentium (691816.5)	1
1235787.3	Bacteroides	Bacteroides uniformis	Bacteroides uniformis dnLKV2	0.9446368
1263055.3	Bacteroides	Bacteroides uniformis	Bacteroides uniformis CAG:3	0.9460181
411479.10	Bacteroides	Bacteroides uniformis	Bacteroides uniformis ATCC 8492	0.9472257
457393.3	Bacteroides	Bacteroides uniformis	Bacteroides uniformis sp. 4_1_36	0.9459347
585543.3	Bacteroides	Bacteroides uniformis	Bacteroides uniformis sp. D20	0.9477446
997889.3	Bacteroides	Bacteroides uniformis	Bacteroides uniformis CL03T00C23	0.9465965
997890.3	Bacteroides	Bacteroides uniformis	Bacteroides uniformis CL03T12C37	0.9454086
